# Angiogenin mediates cell-cell fusion as a mitochondrial RNA processing enzyme

**DOI:** 10.1038/s41413-026-00545-1

**Published:** 2026-06-29

**Authors:** Ke Shen, Yixiang Zeng, Jiekang Wang, Kangping Song, Surendra Kumar, Peisong Gao, Guo-fu Hu, Xu Cao, Mei Wan

**Affiliations:** 1 Department of Orthopaedic Surgery, The Johns Hopkins University School of Medicine, Baltimore, MD USA; 2 Department of Biomedical Engineering, The Johns Hopkins University School of Medicine, Baltimore, MD USA; 3 Division of Allergy and Clinical Immunology, The Johns Hopkins University School of Medicine, Baltimore, MD USA; 4 Division of Hematology and Oncology, Department of Medicine, Tufts Medical Center, Boston, MA USA

**Keywords:** Bone, Pathogenesis

## Abstract

Cell–cell fusion, essential for diverse physiological events, requires high ATP levels. While mitochondrial activity increases in fusing cells, the mechanism driving mitochondrial ribosome (mitoribosome) biogenesis to support these energy demands remains unclear. Here, we identify angiogenin (ANG) as a mitochondrial tRNA (mt-tRNA) processing enzyme critical for mitoribosome biogenesis during myoblast and osteoclast fusion. Upon fusion initiation, ANG translocates to mitochondria, promoting mitoribosome biogenesis to support translation of respiratory complex proteins for ATP production. Using transcriptome-wide PARE and 5′ RACE analyses, we show that ANG cleaves the tRNA 3’-end in mitochondrial pre-RNA transcripts bordering rRNAs and mRNAs, enabling their release for translation. Loss of ANG or disruption of its ribonucleolytic activity impairs osteoclast and myoblast fusion, disrupting bone and muscle homeostasis and skeletal muscle regeneration post-injury. Our findings establish ANG as an essential mitoribosome biogenesis regulator and highlight a novel mechanism of mitochondria energy regulation in high-energy-demand biological processes.

## Introduction

Cell–cell fusion is an evolutionarily conserved biological process occurring in many organisms, including plants, yeast, *C. elegans*, Drosophila, and mammals. In mammals, cell fusion is fundamental for forming multinucleated cells involved in diverse biological events such as myogenesis, osteoclastogenesis, embryonic morphogenesis, placentogenesis, fertilization, and tissue repair.^1–6^ Remarkably, about one-third of cells in animals, from *C. elegans* to humans, have fused with other cells and contain multiple nuclei.^7,8^ Given that cell-cell fusion requires the coordinated rearrangement of both the actin cytoskeleton and fusogenic proteins across the cell membrane,^9–12^ this process is more energy-dependent, necessitating increased mitochondrial biogenesis for ATP production compared to regular cell differentiation.^13–17^ However, despite previous observations of increased number and activity of mitochondria in fusing cells,^18–21^ little is known about how mitoribosome biogenesis is regulated during this specific stage to meet the high energy demands.

Mitochondrial biogenesis is a tightly coordinated process, in which the mitochondrial genome is regulated and expressed in a unique manner, allowing individual mitochondria to respond to changes in membrane potential and maintain oxidative phosphorylation (OXPHOS).^22^ Mammalian mitochondria encodes 2 rRNA genes, 22 tRNA genes, and 13 mRNA genes, the latter coding for membrane proteins required for OXPHOS.^22–24^ The mitochondrial genome itself is transcribed as polycistronic RNA containing rRNAs and mRNAs, which are interspersed by tRNAs in a “punctuation model”, whereby the tRNAs are targeted and cleaved by nuclear-encoded endoribonucleases to release gene products.^25,26^ The mitochondrial tRNA (mt-tRNA) processing involves cleavage at the 5’-end of tRNAs by the RNase P complex^27,28^ and cleavage of the 3’-end by the mitochondrial RNase Z.^29–31^ This canonical processing step is followed by maturation of the RNAs, the assembly of the rRNAs into mitoribosomes, and the translation of the mRNAs. mtRNA processing deficits in mammals cause profound impairments of mitochondrial gene expression, mitochondrial bioenergetics, and cellular/tissue homeostasis, leading to a wide range of disorders across various tissues.^32–36^ Despite advancements in our understanding of mitochondrial RNA processing during homeostasis, it remains unclear whether additional nuclear-encoded endonucleases and exonucleases are required to process larger amount of mt-tRNAs specifically during periods of high energy demand, such as cell-cell fusion.

Given the central role of cell-cell fusion in skeletal muscle health and bone remodeling, myoblasts and osteoclasts are ideal model systems for exploring the regulatory mechanisms of mitoribosome biogenesis in this process. Muscle formation occurs at various life stages, including embryonic development, growth, and adult regeneration. Myogenesis begins with the commitment of satellite cells, muscle stem/progenitor cells, to the myoblast lineage, followed by differentiation of myoblasts into myocytes and myotubes.^37–39^ A critical step in myogenesis is the fusion of myoblasts to form or regenerate multinucleated myofibers, essential for muscle growth, repair, and adaptations to exercise.^40–42^ Similarly, osteoclasts, derived from bone marrow monocytes/macrophages (Mo/Mac), undergo successive cell fusion to form mature multinucleated cells in response to receptor activator of nuclear factor kappa-B ligand (RANKL) stimulation.^43,44^ The number of nuclei generated from these fusion events directly correlates with their bone-resorptive ability.^45,46^ Dysregulation of myoblast and osteoclast fusion leads to multiple musculoskeletal disorders such as muscle weakness,^1,47^ muscular dystrophy, impaired muscle repair/regeneration, dysregulation of bone remodeling, and osteopetrosis.^1,48^

To uncover the molecular mechanisms driving active mitoribosome biogenesis during cell fusion, we utilized myoblasts and osteoclasts as model systems and examined the dynamic changes in mitochondrial biogenesis throughout myoblastogenesis and osteoclastogenesis. Our results revealed increased activation of mitoribosomal biogenesis during cell-cell fusion compared to pre- and post-fusion stages. We identified angiogenin (ANG), a member of the ribonuclease A (RNase A) superfamily, as a novel mt-tRNA processing enzyme required for mitoribosome biogenesis specifically during cell fusion. Loss of ANG impairs mitoribosome biogenesis in myoblast and osteoclast precursors, leading to defective cell fusion, inhibited osteoclastogenesis and myogenesis, as well as impaired bone remodeling and muscle dysfunction.

## Results

### Fusing cells exhibit robustly increased mitochondrial activity

**Fig. 1:**
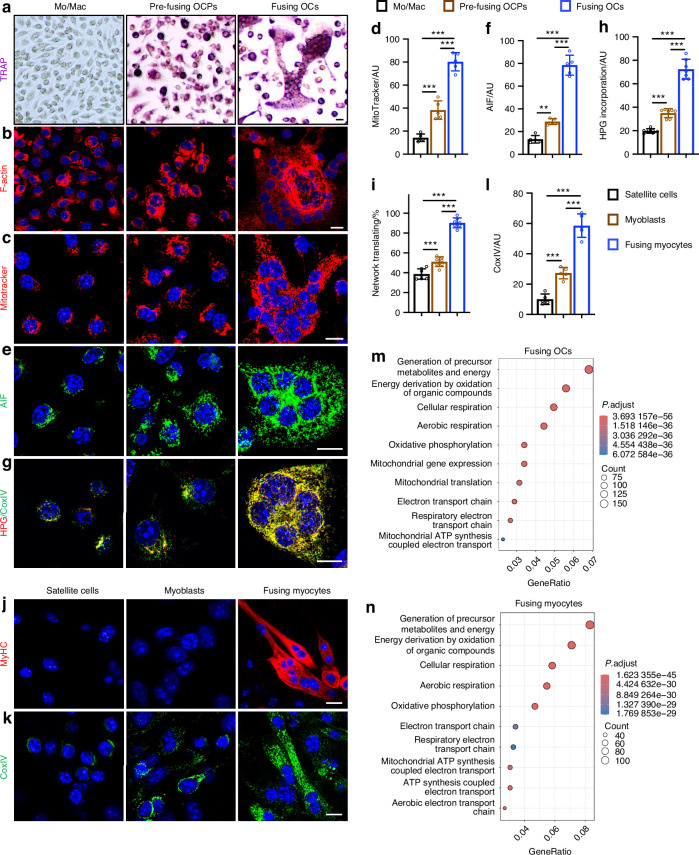
Mitochondrial biogenesis is robustly activated during cell-cell fusion. **a**–**f** Mouse bone marrow monocytes/macrophages (Mo/Mac) cultured in the presence of M-CSF (30 ng/mL) were treated with RANKL (100 ng/mL) for 2 days to acquire pre-fusing osteoclast precursors (Pre-fusing OCPs) and for 3 days to acquire fusing osteoclasts (Fusing OCs). Representative images of the TRAP^+^ cells (**a**) and F-actin rings (**b**). Scale bars, 20 μm. Representative fluorescence imaging of MitoTracker red (**c**) and quantification of the fluorescence intensity (**d**). Scale bar, 20 μm. *n* = 5 samples per group, and 3 fields per sample were calculated. Representative images of immunofluorescence staining of the cells with antibody against AIF (**e**) and quantification of the fluorescence intensity (**f**). Scale bar, 10 μm. *n* = 5 samples per group, and 3 fields per sample were calculated. Representative Mito-FUNCAT images (**g**) of CoxIV (Green) and HPG (Red), and quantification of total HPG incorporation (**h**) as an indicator of mitochondrial protein synthesis, along with the proportion of the mitochondrial network labeled with HPG (**i**), reflecting the translationally active mitochondrial reticulum. Scale bar, 10 μm. *n* = 7 samples per group, and 3 fields per sample were calculated. **j**–**l** Mouse skeletal muscle satellite cells were cultured in the presence of 2% horse serum for 3 days to acquire myoblasts and for 5 days to acquire fusing myocytes. **j** Representative images of the MyHC^+^ cells. Scale bar, 20 μm. Representative fluorescence imaging of CoxIV (**k**) and quantification of the fluorescence intensity (**l**). Scale bar, 20 μm. *n* = 5 samples per group, and 3 fields per sample were calculated. The reanalysis of the published cultured osteoclasts scRNA-seq dataset (GSE147174) was performed. The top 10 GO terms enriched in Fusing OCs and Fusing myocytes were shown in **m**, **n**, respectively. Data are shown as mean ± s.d. and analyzed by one-way ANOVA with Tukey’s multiple comparisons between the indicated groups. ***P < 0.001, and **P < 0.01

We investigated whether mitochondrial biogenesis is activated during cell-cell fusion in osteoclastogenesis and myogenesis. Osteoclastogenesis was induced in vitro by treating bone marrow monocytes/macrophages (Mo/Mac) with RANKL over varying time periods, resulting in the sequential formation of TRAP^+^ mononucleated osteoclast precursors (Pre-fusing OCPs) and TRAP^+^ fusing OCs (Fig. 1a), accompanied by actin belt formation (Fig. 1b). During this process, mitochondrial content, as indicated by MitoTracker Red (Fig. 1c, d) and apoptosis-inducing factor (AIF) (Fig. 1e, f), gradually increased, reaching a peak in fusing OCs. Compared to Mo/Mac, MitoTracker Red levels were only 1.68-fold higher in pre-fusing OCPs but increased 4.62-fold in fusing OCs. Similarly, AIF levels showed a 1.18-fold increase in pre-fusing OCPs but rose significantly, by 4.92-fold, in fusing OCs. A similar trend was observed during myogenesis, where CoxIV^+^ mitochondria were markedly elevated in MyHC^+^ fusing myocytes compared to MyHC^-^ satellite cells and MyHC^+^ single-nuclear pre-fusing myoblasts (Fig. 1j–l), suggesting robust mitochondrial activation during myoblast fusion.

Mitochondrial biogenesis is tightly coupled to mitochondrial translational control.^49,50^ To directly visualize mitochondrial protein synthesis during osteoclast differentiation, we employed a modified mitochondrial fluorescence non-canonical amino acid tagging (Mito-FUNCAT) strategy.^51,52^ This approach labels newly synthesized polypeptides with the methionine analog L-homopropargylglycine (HPG) and selectively reports mitochondrial translation by inhibiting cytosolic protein synthesis with cycloheximide. Consistent with the marked upregulation of AIF in fusion stage (Fig. 1e, f), fusing OCs exhibited substantially increased mitochondrial HPG incorporation and a higher proportion of translationally active mitochondrial networks compared with Mo/Mac and pre-fusing OCPs (Fig. 1g–i), which indicated that enhanced mitochondrial protein synthesis supports the fusion during osteoclast differentiation.

To verify the above results, we re-analyzed publicly available single-cell RNA-sequencing (scRNA-Seq) datasets that map the sequential steps of osteoclastogenesis and myogenesis. We first examined a dataset from murine bone marrow cells undergoing stepwise in vitro osteoclastogenesis (GSE147174)^53^ by reclassifying osteoclast lineage cells into distinct clusters corresponding to 4 differentiation stages: bone marrow Mo/Mac in the absence of RANKL stimulation (Mo/Mac); early osteoclast precursors (Early OCPs); pre-fusing osteoclast precursors (Pre-fusing OCPs); and fusing osteoclasts (Fusing OCs) (Fig. S1a). Pseudotime analysis of the differentiation trajectory of osteoclast lineage revealed a stepwise pathway where Mo/Mac differentiated into fusing OCs, passing through early OCPs and Pre-fusing OCPs (Fig. S1b, c). During this process, genes associated with undifferentiated Mo/Mac (*Fn1*, *Ccr2*, and *F13a1*) were gradually downregulated, while genes associated with osteoclast differentiation, including *Tnfrsf11a*, *NFATc1*, and *Acp5*, and *Ctsk* were progressively upregulated (Fig. S1d). Osteoclast fusion-associated genes *Ocstamp* and *Dcstamp* were highly expressed, while *Mki67* expression was nearly absent in Fusing OCs, confirming that Pre-fusing OCPs are non-fused cells, and Fusing OCs are actively undergoing fusion (Fig. S1d). Gene ontology (GO) enrichment analysis revealed that processes enriched in pre-fusing OCPs were related to rRNA transcription and processing, which occur predominantly in the cell nucleus (Fig. S2a). Consistent with our immunostaining results, Fusing OCs displayed strong enrichment in pathways closely linked to mitochondrial activities, including OXPHOS, mitochondrial gene expression, and cell respiration (Fig. 1m). Monocle 3 analysis further revealed progressive upregulation of mitochondrial genes during osteoclast differentiation, peaking at the fusing OC stage (Fig. S2b).

We also analyzed a dataset from muscle satellite cells and primary myoblasts isolated from homeostatic/regenerating muscles (GSE126834).^54^ Using established marker genes from previous benchmark studies,^54–56^ we reclassified myoblast lineage cells into four distinct cell clusters corresponding to differentiation stages: Satellite cells, myoblasts, fusing myocytes, and myotubes (Fig. S3a). Specifically, *Pax7*^*low*^*/Myf5*^*hi*^ cells were considered as early-activated satellite cells, while *Myf5*^*low*^*Myog*^*low*^*/Myod1*^*hi*^*Ccnd1*^*hi*^*Ccnd2*^*h*i^ cells represented pre-fusing myoblasts. Cells enriched in *Myog* and myoblast fusion factors *Mymx* and *Mymk*, but lacking creatine kinase *Ckm* and the myofiber transcripts *Myh1* and *Myh7* were classified as fusing myocytes. Cells enriched in *Acta1*, *Ckm*, *Myh1*, and *Myh7* were identified as mature myotubes (Fig. S3b). Trajectory analysis revealed a sequential differentiation pathway, with satellite cells transitioning into mature multinucleated myotubes through intermediate stages of myoblasts and fusing myocytes (Fig. S3c). GO analysis demonstrated that similar to fusing OCs, fusing myocytes are primarily associated with mitochondrial activities, including cell respiration, OXPHOS, and mitochondrial ATP synthesis coupled electron transport (Fig. 1n). In contrast, earlier stage non-fusing satellite cells, myoblasts, and mature myotubes were much less associated with mitochondria function (Fig. S4), suggesting a robust increase in mitochondrial biogenesis to meet the higher energy demands of cell fusion.

### ANG is transported to mitochondria during cell-cell fusion

We next sought to identify key molecular changes associated with mitochondria during cell-cell fusion. KEGG functional enrichment analysis demonstrated that pathways related to neurodegenerative diseases, including amyotrophic lateral sclerosis (ALS), Alzheimer’s disease (AD), and Parkinson’s disease (PD), were prominently enriched alongside OXPHOS in fusing myocytes (Fig. S5a). Among the candidates, *ANG*, also known as ribonuclease 5 (RNase 5), drew our attention due to its upregulation in fusing myocytes compared to earlier-stage satellite cells and its marked downregulation in later stage myotubes (Fig. S5b). Moreover, loss of function mutations in *ANG* are associated with all three of these neurodegenerative diseases: ALS,^57,58^ AD,^59,60^ and PD.^60,61^ Notably, the ANG protein contains an internal matrix targeting-like sequence (iMTS-Ls) (Fig. S6), which is known to direct proteins from the cytosol to mitochondria,^62^ suggesting its possible involvement in mitoribosomal biogenesis.

**Fig. 2:**
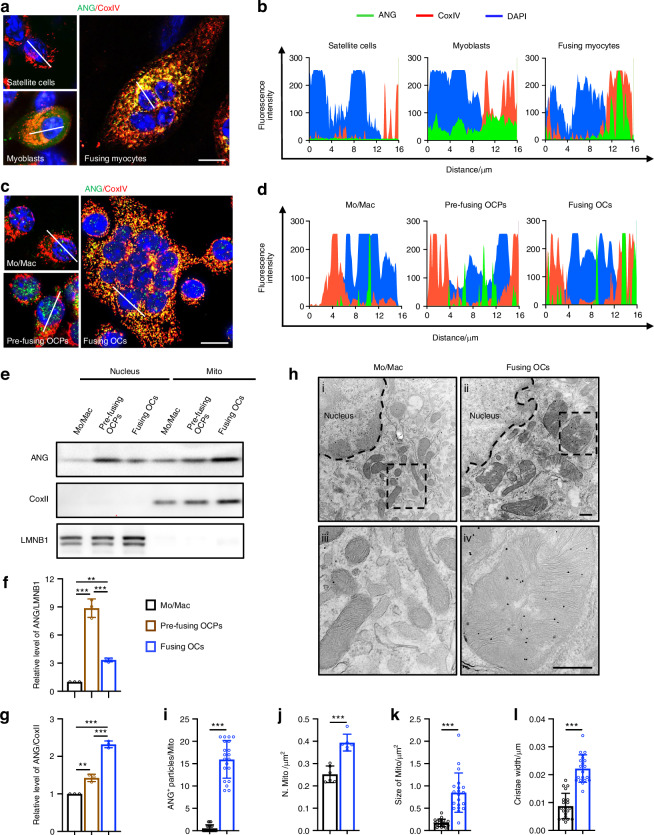
ANG is robustly transported to mitochondria during cell-cell fusion. **a**, **b** Mouse skeletal muscle satellite cells were cultured in the presence of 2% horse serum for 3 days to acquire myoblasts and for 5 days to acquire fusing myocytes. Representative images of double-immunofluorescence staining of the cells appeared in each differentiation stage during myogenesis with antibodies against ANG and CoxIV (**a**). Scale bars, 20 μm. Measurements of the fluorescence intensity of the cells across a line segment spanning individual cells in **a** were shown in (**b**). **c**, **d** Bone marrow Mo/Mac cultured in the presence of M-CSF (30 ng/mL) were treated with RANKL (100 ng/mL) for 2 days to acquire pre-fusing OCPs and for 3 days to acquire fusing OCs. Representative images of double-immunofluorescence staining of the cells appeared in each differentiation stage during osteoclastogenesis with antibodies against ANG and CoxIV (**c**). Scale bars, 20 μm. Measurements of the fluorescence intensity of the cells across a line segment spanning individual cells in **c** were shown in (**d**). **e**–**g** Bone marrow Mo/Mac cultured in the presence of M-CSF (30 ng/mL) were treated with RANKL (100 ng/mL) for 2 days to acquire pre-fusing OCPs and for 3 days to acquire fusing OCs. Western blot analysis of protein expression of ANG, mitochondrial protein CoxII, and nuclear protein LMNB1 using protein extracted from nucleus and mitochondria (Mito) during osteoclast differentiation (**e**). Quantify and normalize ANG in the nucleus relative to LMNB1 (**f**), and in the mitochondria relative to CoxII (**g**), respectively. **h**–**l** Bone marrow Mo/Mac cultured in the presence of M-CSF (30 ng/mL) were treated with RANKL (100 ng/mL) for 3 days to acquire fusing OCs or left untreated. Localization of ANG in Mo/Mac and fusing OCs by immunogold labeling in electron microscopy. Immunostaining was performed with antibodies against ANG conjugated with gold particles (black dots) (**h**). Quantified analyses of the number of ANG positive particles per mitochondria (**i**), the number of mitochondria per μm^2^ area (**j**), the size of mitochondria (**k**) and the width of cristae (**l**) were calculated. Scale bars, 500 nm. *n* = 6 samples per group for **j**, and 20 mitochondrion per group for **i**, **k**, **l** were calculated. Data are shown as mean ± s.d. and analyzed by unpaired Student’s *t* test between the indicated groups. ***P < 0.001, and **P < 0.01

We examined the dynamic changes of subcellular distribution of ANG during each step of the myogenesis and osteoclastogenesis process. While ANG expression was almost undetectable in satellite cells, pre-fusing myoblasts and fusing myocytes have much more abundant ANG expression (Fig. 2a, b). Intriguingly, while ANG was detected in both nucleus and cytosol in pre-fusing myoblasts, ANG was exclusively co-localized with mitochondria marker CoxIV in fusing myocytes (Fig. 2a, b). Similarly, in osteoclast lineage cells, ANG expression increased in the nucleus in pre-fusing OCPs compared to Mo/Mac. Fusing OCs showed a sharp increase in ANG expression, which primarily co-localized with the mitochondrial marker CoxIV (Fig. 2c, d). These results suggest that ANG is translocated into mitochondria during both myoblast and osteoclast fusion. To verify the translocation of ANG from the nucleus to the mitochondria during osteoclastogenesis, we conducted subcellular fractionation of cells at each step of the osteoclast differentiation process. Consistent with the immunofluorescence staining results, western blot analysis revealed low levels of ANG in both the nucleus and mitochondria in Mo/Mac. However, in pre-fusing OCPs, ANG level increased significantly in the nucleus; and in fusing OCs, it increased notably in the mitochondria (Fig. 2e–g). Additionally, we used immune-electron microscopy to examine the submitochondrial localization of ANG. In Mo/Mac, only a few scattered ANG gold particles were observed in the nucleus and cytosol, with almost no detectable gold particles in the mitochondria. Strikingly, in fusing OCs, a significantly higher number of gold particles were present in the mitochondria, primarily associated with the mitochondrial matrix and inner membrane (Fig. 2h, i). Of note, besides the increase in the number of mitochondria (Fig. 2j), mitochondria in fusing OCs (Fig. 2h-ii, iv) were much larger than those in Mo/Mac (Fig. 2h-i, iii). The average size of the small mitochondria in Mo/Mac was 0.17 µm^2^, whereas the average size of the “giant” mitochondria was 0.85 µm^2^ with the presence of shelf-like cristae structures (Fig. 2k, l).

### ANG is a positive regulator of cell-cell fusion

**Fig. 3:**
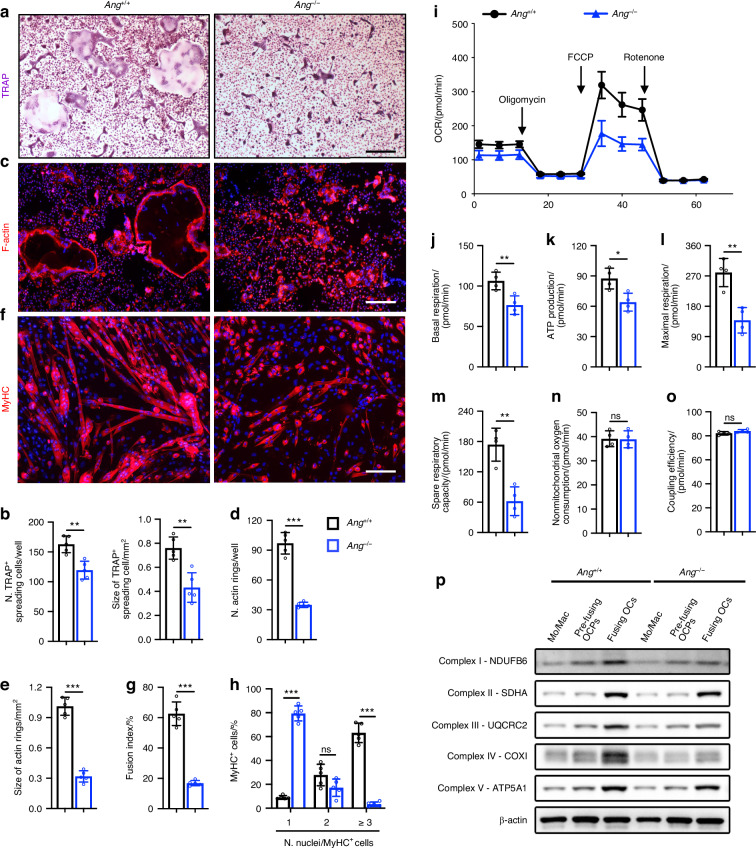
Loss of ANG induces mitochondrial dysfunction in fusing cells. **a**–**e** Bone marrow Mo/Mac were isolated from 1-month-old *Ang* deficient (*Ang*^ − /−^) mice and their wild-type littermates (*Ang*^ + /+^) and cultured in the presence of M-CSF (30 ng/mL) and RANKL (100 ng/mL) for 4 days to acquire mature osteoclasts. TRAP staining and F-actin staining were performed. Representative images were shown in **a**, **c** respectively. Scale bars, 200 μm. Quantified analyses of the number and size of TRAP^+^ spreading cells per well were shown in (**b**). Quantified analyses of the number and the size of actin rings per well were shown in **d**, **e**, respectively. *n* = 5 samples per group, and 3 fields per sample were calculated. **f**–**h** Skeletal muscle satellite cells were isolated from 2-month-old *Ang*^ − /−^ and *Ang*^ + /+^ mice. Muscle differentiation were induced in the presence of 2% horse serum for 7 days of to acquire mature myotubes. MyHC staining was performed. Representative images were shown in (**f**). Scale bar, 50 μm. Quantified analyses of the fusion index and the percentages of cells with different numbers of nuclei were shown in **g**, **h**, respectively. *n* = 5 samples per group, and 3 fields per sample were calculated. **i**–**o** Bone marrow Mo/Mac from *Ang*^ + /+^ and *Ang*^ − /−^ mice were treated with M-CSF (30 ng/mL) and RANKL (100 ng/mL) for 3 days to acquire fusing OCs. Oxygen consumption rates (OCR) (**i**), Basal respiration (**j**), ATP production (**k**), maximal respiratory capacity (**l**), spare respiratory capacity (**m**), Nonmitochondrial oxygen consumption (**n**) and coupling efficiency (**o**) were assessed by Seahorse respiration phenotype measurements. *n* = 4 mice per group. **p** Bone marrow Mo/Mac from *Ang*^ + /+^ and *Ang*^ − /−^ mice were treated with M-CSF (30 ng/mL) and RANKL (100 ng/mL) for 0, 2, or 3 days to acquire Mo/Mac, pre-fusing OCPs, and fusing OCs, respectively. Western blot analyses of the indicated mitochondria proteins were shown. β-actin expression serves as loading control. Data are shown as mean ± s.d. and analyzed by unpaired Student’s *t* test or two-way ANOVA with Tukey’s multiple comparisons between the indicated groups. ***P < 0.001, **P < 0.01, *P < 0.05, and ns ≥ 0.05

As ANG translocates into mitochondria during cell fusion, we examined if ANG plays a role in osteoclast and myoblast fusion. We first assessed the changes of osteoclastogenesis and myogenesis using ANG-deficient cells isolated from *Ang* null mice. Osteoclastogenesis was stimulated by treating Mo/Mac from *Ang* knockout (*Ang* ^− /−^) mice and their wild-type littermates (*Ang*^ + /+^) with RANKL. Osteoclastogenesis was markedly impaired in cells from *Ang*^ − /−^ mice compared with those from *Ang*^ + /+^ mice, as evidenced by a reduced number and size of multinucleated osteoclasts (Fig. 3a, b) and defective actin ring formation (Fig. 3c–e). Bone-resorptive activity of the *Ang*^ − /−^ osteoclasts was also disrupted, as observed in the resorption pit assays (Fig. S7a, b). To test the role of ANG in myogenesis, satellite cells isolated from *Ang*^ + /+^ and *Ang*^ − /−^ mice were induced for differentiation. *Ang*^ − /−^ cells exhibited a diminished capacity to form MyHC^+^ myotubes (Fig. 3f). The fusion index (Fig. 3g) and the number of nuclei per MyHC^+^ cells (Fig. 3h) were dramatically reduced in *Ang*^ − /−^ cells compared to *Ang*^ + /+^ cells. The results suggest that ANG is a key positive regulator of cell-cell fusion and is therefore essential for the differentiation process in both cell lineages.

### Loss of ANG impairs mitoribosome biogenesis in fusing cells

We then assessed whether the impaired cell-cell fusion in *Ang*^ − /−^ cells was attributed to mitochondrial dysfunction. As expected, there was an overall reduction in oxygen consumption rates (OCRs) in *Ang*^ − /−^ cells compared with *Ang*^ + /+^ cells during osteoclast differentiation (Fig. 3i). Basal respiration rate (Fig. 3j) and ATP-linked respiration (Fig. 3k) were notably lower in the *Ang*^ − /−^ cells compared with the *Ang*^ + /+^ cells, reflecting a reduced capacity of *Ang*^ − /−^ cells to use membrane potential as a driving force for ATP synthesis. Furthermore, maximal respiration (Fig. 3l) and spare respiration capacity (Fig. 3m) were diminished in *Ang*^ − /−^ cells relative to *Ang*^ + /+^ cells, indicating impaired reserve capacity of the ANG-deficient cells for ATP generation via OXPHOS under the condition of increased energy demand. However, nonmitochondrial oxygen consumption (Fig. 3n) and coupling efficiency (Fig. 3o) were not significantly different between *Ang*^ − /−^ and *Ang*^ + /+^ cells, suggesting that nonmitochondrial respiration and mitochondrial efficiency of substrate oxidation were unaffected by ANG deficiency.

**Fig. 4:**
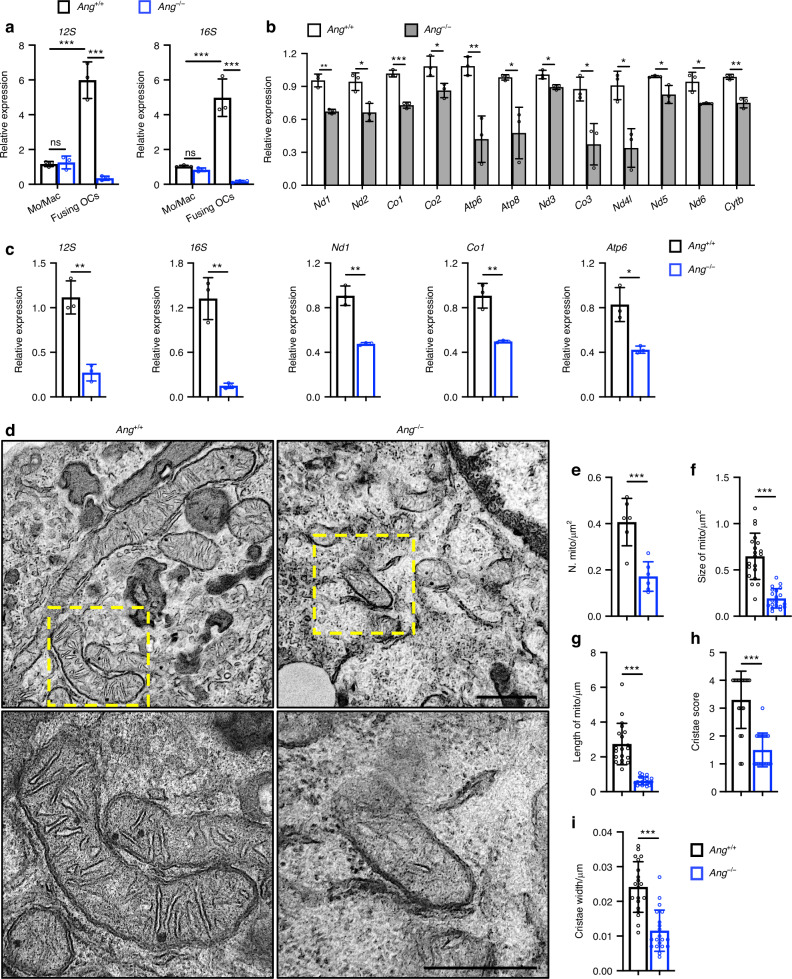
Loss of ANG impairs mitoribosome biogenesis in fusing cells. **a**, **b** Bone marrow Mo/Mac from *Ang*^ + /+^ and *Ang*^ − /−^ mice were treated with M-CSF (30 ng/mL) and RANKL (100 ng/mL) for 3 days to acquire fusing OCs or left untreated. Real time-qPCR measurement of mitochondrial *12S* rRNAs (**a** left panel), *16S* rRNAs (**a** right panel), and mitochondrial mRNAs (**b**). *n* = 3 samples per group. **c** Skeletal muscle satellite cells from *Ang*^ + /+^ and *Ang*^ − /−^ mice were treated with 2% horse serum for 5 days to acquire fusing myocytes. Real time-qPCR measurements of mitochondrial rRNAs and mRNAs were shown. *n* = 3 samples per group. **d**–**i** Bone marrow Mo/Mac from *Ang*^ + /+^ and *Ang*^ − /−^ mice were treated with M-CSF (30 ng/mL) and RANKL (100 ng/mL) for 3 days to acquire fusing OCs. Representative electron microscopy images of fusing OCs (**d**) with quantifications of mitochondria numbers (**e**), mitochondria area (**f**), mitochondria length (**g**), cristae score (**h**), and cristae width (**i**). Scale bars, 500 nm. *n* = 6 samples/group for **e** and 20 mitochondrion/group for **f**–**i** were calculated. Data are shown as mean ± s.d. and analyzed by unpaired Student’s *t* test between the indicated groups. ***P < 0.001, **P < 0.01, *P < 0.05, and ns ≥ 0.05

To elucidate the molecular basis of the observed respiration phenotype, we evaluated the levels of electron-translocating respiratory complex components by western blotting. There was a progressive increase in the levels of both nuclear- and mitochondrial-DNA encoded components in *Ang*^ + /+^ cells during osteoclast differentiation (Fig. 3p), indicating robust activation of protein translation machinery during this process. While certain nucleus-encoded subunits, such as the complex II component SDHA, remained unchanged, the mtDNA-encoded complex IV components CoxI and CoxII were notably diminished in *Ang*^ − /−^ cells compared with *Ang*^ + /+^ cells in fusing OCs) (Fig. 3p and Fig. S8). NDUFB6, a nucleus-encoded complex I subunit whose assembly relies on the presence of the mtDNA-encoded ND4 protein,^63^ was also reduced (Fig. 3p and Fig. S8). These results suggest that loss of ANG impairs the biogenesis of electron-translocating respiratory complexes, thereby limiting their formation. Consistently, while expression levels of mitochondrial ribosomal RNA transcripts *12S* rRNA and *16S* rRNA were dramatically increased in *Ang*^ + /+^ cells when osteoclast fusion occurs, the expression levels of these two rRNAs were not elevated in *Ang*^ − /−^ cells in response to RANKL (Fig. 4a). Further, all mitochondria-encoded genes that were elevated in *Ang*^ + /+^ cells during fusion were markedly reduced in *Ang*^ − /−^ cells (Fig. 4b, c). We also assessed mitochondrial morphology changes in ANG-deficient cells using transmission electron microscopy. Both the number of mitochondria (Fig. 4d, e) and the area occupied by mitochondria (Fig. 4d, f) were significantly lower in *Ang*^ − /−^ cells compared with *Ang*^ + /+^ cells during osteoclast fusion. Whereas *Ang*^ + /+^ cells exhibited normally shaped mitochondria with multiple cristae, *Ang*^ − /−^ mitochondria displayed aberrant morphology, including smaller size (Fig. 4g), fewer cristae (Fig. 4h), and reduced cristae width (Fig. 4i). Overall, these results demonstrate that loss of ANG results in severe mitochondrial dysfunction during osteoclastogenesis and myogenesis due to impaired mitoribosome biogenesis and diminished ATP-linked oxygen consumption and respiration.

### ANG-deficient mice exhibit muscle and bone defects

**Fig. 5:**
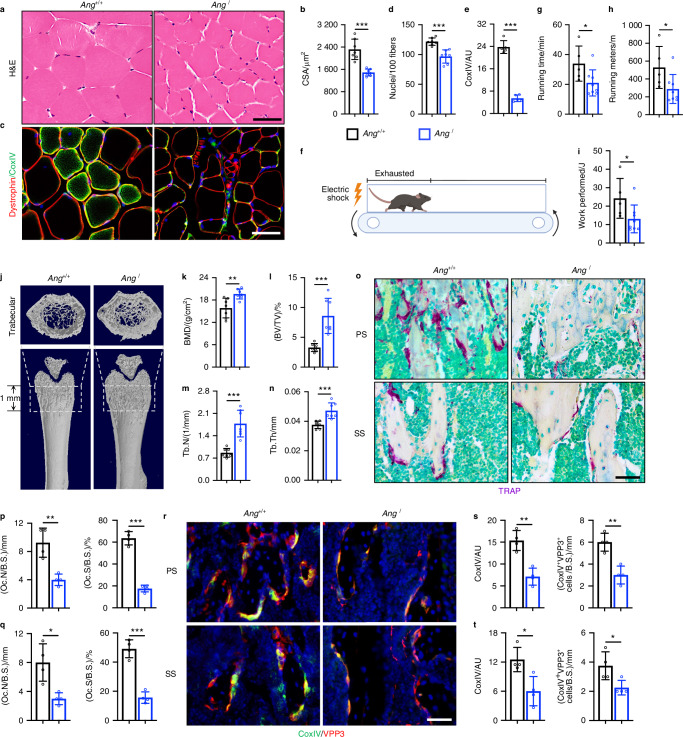
ANG is essential to maintain muscle and bone homeostasis. **a** The GA muscles were harvested from 4-month-old *Ang* deficient (*Ang*^ − /−^) mice and wild-type littermates (*Ang*^ + /+^). Representative images of H&E staining and double-immunofluorescence staining with antibodies against ANG and CoxIV were shown in **a**, **c**, respectively. Scale bars, 50 μm. Quantification of the muscle fiber cross-sectional area (CSA) (**b**), myonuclei per 100 myofibers (**d**), and CoxIV fluorescence intensity (**e**). *n* = 6 samples per group and 3 fields per sample were calculated. **f**–**i** Exercise performance was evaluated in 4-month-old *Ang*^ + /+^ and *Ang*^ − /−^ mice using a treadmill exhaustion test (**f**). Quantitative measurements included time to exhaustion (**g**), running distance (**h**), and work performed (**i**) during the test. *n* = 5 for *Ang*^ + /+^ mice and *n* = 8 for *Ang*^ − /−^ mice. **j**–**n** Micro-CT analyses of the distal femurs in 1-month-old *Ang*^ + /+^ and *Ang*^ − /−^ mice. Representative 3D-reconstruction images were shown in (**j**). Top: trabecular cross sections, and bottom: longitudinal sections. Quantifications of bone mineral density (BMD) (**k**), trabecular bone volume fraction (BV/TV) (**l**), trabecular number (Tb. N) (**m**), and trabecular thickness (Tb. Th) (**n**). *n* = 7 mice per group. **o**–**q** TRAP staining of femoral bone tissue sections prepared from 1-month-old *Ang*^ + /+^ and *Ang*^ − /−^ mice. Representative images were shown in (**o**). PS: primary spongiosa; SS: secondary spongiosa. Osteoclast number (Oc.N/B.S.) and osteoclast surface (OS/B.S.) per bone surface were quantified in PS (**p**) and SS (**q**). Scale bars, 50 μm. **r**–**t** Double-immunofluorescence staining of bone tissue sections using antibodies against CoxIV and VPP3. Representative images are shown in (**r**). CoxIV fluorescence intensity and CoxIV^+^VPP3^+^ double positive cells were calculated in PS (**s**) and SS (**t**). Scale bars, 50 μm. *n* = 4. Data are shown as mean ± s.d. and analyzed by unpaired Student’s *t* test. ***P < 0.001, **P < 0.01, and *P < 0.05

We next examined whether ANG deficiency-induced defects in cell-cell fusion lead to dysregulated skeletal muscle and bone homeostasis. The wet weight of the gastrocnemius (GA) muscles harvested from 4-month-old *Ang*^ − /−^ mice was reduced compared to that of age-matched *Ang*^ + /+^ mice (Fig. S9). H&E staining revealed a significant decrease in average myofiber cross-sectional area (CSA) in the GA of *Ang*^ − /−^ mice relative to *Ang*^ + /+^ mice (Fig. 5a, b). Dystrophin staining demonstrated fewer myonuclei in dystrophin^+^ muscle fibers in *Ang*^ − /−^ mice compared to *Ang*^ + /+^ mice (Fig. 5c, d). Additionally, numerous unfused mononucleated satellite cells were observed in the GA of Ang-deficient mice, potentially impairing muscle function (Fig. 5c). Co-immunofluorescence staining of muscle tissue sections with dystrophin and CoxIV revealed a much weaker CoxIV^+^ signal in *Ang*^ − /−^ mice (Fig. 5c, e), indicating lower mitochondrial content in ANG-deficient muscles. We also assessed muscle functional changes by performing treadmill exhaustion test (Fig. 5f). *Ang*^ − /−^ mice had shorter times to exhaustion (Fig. 5g), reduced running distances (Fig. 5h), and a 45.98% reduction in total work performed (Fig. 5i) compared to their *Ang*^ + /+^ littermates.

We also assessed the bone phenotypic changes of both young and adult ANG deficient mice. At 1 month of age, *Ang*^ − /−^ mice exhibited a high bone mass phenotype (Fig. 5j). Micro-CT analysis of trabecular bone revealed a significant increase in bone mineral density (BMD) (Fig. 5k), bone volume per tissue volume (BV/TV) (Fig. 5l), trabecular number (Tb.N) (Fig. 5m), and trabecular thickness (Tb.Th) (Fig. 5n) in *Ang*^ − /−^ mice compared with *Ang*^ + /+^ mice. Similar trabecular changes were observed in 4-month-old adult *Ang*^ − /−^ mice relative to *Ang*^ + /+^ mice (Fig. S10a–d). Histological analysis of bone tissue sections revealed a significant decrease in TRAP^+^ osteoclasts with markedly lower osteoclast number (Oc.N/mm) and surface (Oc.S/B.S.%) in both primary spongiosa (PS) (Fig. 5o upper panel, and 5p) and secondary spongiosa (SS) (Fig. 5o lower panel, and 5q) regions of the distal femur in 1-month-old *Ang*^ − /−^ mice compared with *Ang*^ + /+^ mice. Of note, the sizes of bone surface osteoclasts appeared much smaller in *Ang*^ − /−^ mice relative to *Ang*^ + /+^ mice (Fig. 5o). Similarly, diminished bone surface osteoclasts were also observed in 4-month-old *Ang*^ − /−^ mice relative to *Ang*^ + /+^ mice (Fig. S10e–g).

We next evaluated whether ANG deficiency-induced impairment of osteoclast fusion affects bone regeneration using a mouse drill-hole cortical defect model, a widely used and highly reproducible approach for studying bone fracture healing.^64–67^ In this model, mineralized woven bone is present within the drill site by day 7 post-injury, and the woven bone undergoes active remodeling by day 14 after surgery.^64–67^ Micro-CT analysis revealed that *Ang*^ − /−^ mice exhibited significantly greater mineralized bone within the healing defect (Fig. S11a) and an increased (BV/TV)/% (Fig. S11b) compared with *Ang*^ + /+^ mice. TRAP staining showed a significant reduction in osteoclast number and activity and at the woven bone site (Fig. S11c, d), indicating impaired active remodeling of newly formed bone. Consistently, H&E staining of bone sections revealed excessive and enlarged woven bone with a disorganized bone structure in the healing sites of *Ang*^ − /−^ mice relative to *Ang*^ + /+^ controls (Fig. S11e, f). The results suggest that the initial woven bone formed within the defect is not efficiently replaced by mature bone due to defective osteoclast-mediated callus remodeling.

We then examined whether ANG deficiency impairs mitochondria activity in osteoclast by performing double-immunofluorescence staining of bone sections from *Ang*^ − /−^ mice and *Ang*^ + /+^ controls. Both the fluorescence intensity of CoxIV and the number of CoxIV^+^VPP3^+^ double-positive osteoclasts were markedly reduced in the PS (Fig. 5r upper panel, and 5s) and SS (Fig. 5r lower panel, and 5t) in *Ang*^ − /−^ mice relative to *Ang*^ + /+^ mice, suggesting mitochondrial dysfunction in osteoclasts in vivo. These in vivo data suggest that ANG-associated mitochondrial function is critical for maintaining muscle and bone homeostasis. Loss of ANG leads to mitochondrial dysfunction, ultimately resulting in muscle dysfunction and defective bone modeling.

Our previous studies demonstrated that osteoclast-derived ANG maintains the homeostasis of type-H vessels in metaphysis of growing young mice.^68^ To determine whether mitochondrial activity is also impaired in bone vascular cells, we performed multiplex immunofluorescence staining of femoral bone tissue from one-month-old *Ang*^ − /−^ mice, a stage at which type H vessels are abundant and specifically enriched in the metaphysis of long bones.^68,69^ In WT mice, we observed abundant CD31^+^ Emcn^+^ type H vessels accompanied by strong expression of the mitochondria marker CoxIV in the metaphysis region, where ANG is strongly expressed in vessel-associated osteoclasts.^68^ In contrast, both CD31^+^ Emcn^+^ type H vessels and CoxIV expression were significantly reduced in the same region of *Ang*^ − /−^ mice (Fig. S12a–c). The results suggest that type H vessel formation in metaphysis is associated with elevated mitochondrial activity, likely mediated by ANG through a non-cell-autonomous mechanism, given the very low level of ANG expression in bone vascular cells.^68^

### Loss of ANG causes defects in mt-tRNA 3’-end processing in fusing cells

**Fig. 6:**
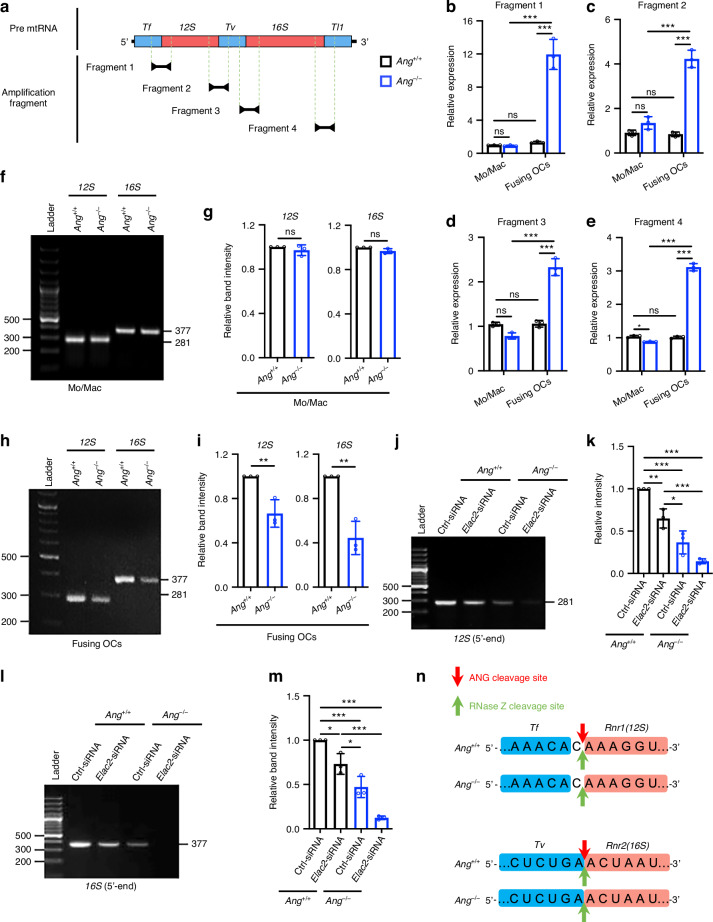
Loss of ANG causes defects in mt-tRNA 3’-end processing in fusing cells. **a** A schematic diagram shows mitochondrial rRNAs and the bordering tRNAs in mitochondrial Pre-RNA. The related positions of the fragments being amplified by RT-qPCR are also depicted. **b**–**e** Bone marrow Mo/Mac cultured in the presence of M-CSF (30 ng/mL) were treated with RANKL (100 ng/mL) for 3 days to acquire fusing OCs or left untreated. Expressions of the four selected fragments indicated in **a** in the cells were measured by qRT-PCR. *n* = 3 mice per group. Mo/Mac from *Ang*^ + /+^ and *Ang*^ − /−^ mice were cultured in medium containing M-CSF (30 ng/mL) with (**h**, **i**) or without (**f**, **g**) RANKL treatment (100 ng/mL for 3 days). Mitochondrial RNA from the cells were subjected to 5’RACE. Agarose gel analysis (**f**, **h**) and associated quantification (**g**, **i**) of 1 µg 5’RACE products of 5’-end of *12S* and *16S*. *n* = 3 mice per group. **j**–**m** Bone marrow Mo/Mac from *Ang*^ + /+^ and *Ang*^ − /−^ mice were transfected with individual siRNAs as indicated followed by RANKL (100 ng/mL) for 3 days to acquire fusing OCs. Mitochondrial RNA from the cells were subjected to 5’RACE. Agarose gel analysis (**j**, **l**) and associated quantification (**k**, **m**) of 1 µg 5’RACE products corresponding to 5’-end of *12S* and *16S*. *n* = 3 mice per group. **n**, A diagram illustrates the 5’-end cleavage patterns of *12S* and *16S*. Data are shown as mean ± s.d. and analyzed by unpaired Student’s *t* test or one-way ANOVA or two-way ANOVA with Tukey’s multiple comparisons between the indicated groups. ***P < 0.001, **P < 0.01, *P < 0.05, and ns ≥ 0.05

We next sought to identify the molecular mechanisms through which ANG regulates mitoribosome biogenesis in fusing cells. The observed 4- to 9-fold increase in levels of *12S* rRNA and *16S* rRNA in WT cells but not in ANG-deficient cells during cell fusion (Fig. 4a, c) suggests a defect in mt-rRNA processing. It is known that rRNAs in mammalian mitochondria are flanked by tRNA genes (Fig. 6a), which are processed by mitochondrial RNase P and RNase Z to release the rRNAs for mitoribosome biogenesis. We measured the levels of *12S* rRNA and *16S* rRNA unprocessed precursors by RT-qPCR analyses of the 4 junction regions between the tRNA and *12S*/*16S* sequences (Fragment 1–4 in Fig. 6a) in *Ang*^ − /−^ cells and *Ang*^ + /+^ cells. In Mo/Mac without RANKL treatment (non-fusing cells), none of the *12S* rRNA-precursor and *16S* rRNA-precursor ends was significantly altered in *Ang*^ − /−^ cells relative to *Ang*^ + /+^ cells. However, all of the *12S* rRNA- and *16S* rRNA-precursor ends, including the 3’- and 5’-end of tRNA molecules, were markedly higher in *Ang*^ − /−^ cells compared with *Ang*^ + /+^ cells by day 3 of RANKL treatment (fusing cells) (Fig. 6b–e), suggesting an accumulation of the unprocessed rRNA precursors when ANG is deficient during cell fusion.

To assess the direct involvement of ANG in mtRNA processing, we first performed 5’RACE experiments. In the absence of RANKL stimulation, *Ang*^ − /−^ Mo/Mac had similar abundance (Fig. 6f, g) and the same size (Fig. S13) of the products in the 5’-end of *12S* rRNA and *16S* rRNA, which borders the 3’-end of mt-tRNAs, relative to *Ang*^ + /+^ Mo/Mac. However, during cell fusion, there were significantly less abundant products in the 3’-end of mt-tRNAs bordering *12S* rRNA and *16S* rRNA in *Ang*^ − /−^ cells compared with *Ang*^ + /+^ cells (Fig. 6h, i), although the sizes of the products appeared the same. Further sequencing the amplification products showed same cleavage sites of the 5’-end of both *12S* and *16S* rRNA in *Ang*^ − /−^ cells relative to *Ang*^ + /+^ cells (Fig. S14). Therefore, there was a markedly less efficient cleavage of the 3’ tRNA cleavage sites that border *12S* and *16S* rRNA upon ANG loss. RNase Z is the only endoribonuclease identified to date that is responsible for the processing of the 3’-end of mt-tRNAs in mammalian cells. To determine whether ANG alone is able to directly cleave 3’-end tRNA or whether it just assists the cleavage activity of RNase Z during osteoclast fusion, we conducted 5’RACE in *Ang*^ − /−^ and *Ang*^ + /+^ cells that were transfected with siRNA to knock down *Elac2* (RNase Z encoding gene) (Fig. S15a). Although the abundance of the product in the 5’-end of *12S* rRNA was slightly lower (35% reduction) in *Elac2* siRNA- vs. control siRNA-transfected WT cells in response to RANKL stimulation (Fig. 6j 2nd lane vs. 1st lane), *Ang* deficiency caused more profound reduction in the abundance of the product in control siRNA- (Fig. 6j 3rd lane vs. 1st lane, and 6k) and *Elac2* siRNA-transfected cells (Fig. 6j 4th lane vs. 2nd lane), leading to 63% reduction. The same result was obtained for the product in the 5’-end of *16S* rRNA (Fig. 6l, m). The cleavage sites of the 5’-end of both *12S* and *16S* rRNA were the same in the cells of all groups when comparing the sequencing data of the amplification products (Fig. S15b). These data suggest that ANG and RNase Z may have identical cleavage sites on mt-tRNAs (Fig. 6n); however, ANG exhibits higher endoribonuclease activity than RNase Z in cleaving the 3’-end of mitochondrial tRNAs bordering *12S* and *16S* rRNAs, specifically during osteoclast fusion.

**Fig. 7:**
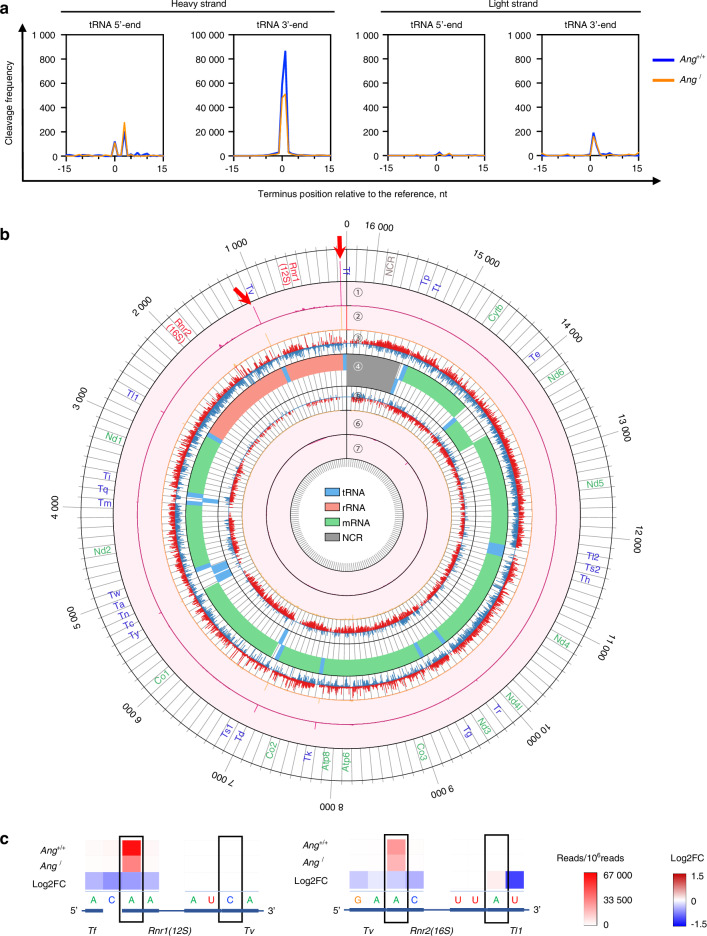
Transcriptome-wide Analyses of 3’-end of tRNA Cleavage Sites by PARE-Seq. **a**, **b** Bone marrow Mo/Mac from *Ang*^ + /+^ and *Ang*^ − /−^ mice were treated with M-CSF (30 ng/mL) and RANKL (100 ng/mL) for 3 days to acquire fusing OCs. Mitochondrial RNA isolated from the cells were subjected to PARE-sequencing. Statistical analysis of the total cleavage frequencies at the 5’-end and 3’-end of all mitochondrial tRNAs from the heavy and light strands was shown in (**a**). The statistical range includes 15 nucleotides (nt) on either side of the intergenic regions. A complete map of the cleavage frequencies (① *Ang*^ + /+^ heavy strand; ② *Ang*^ − /−^ heavy strand; ⑥ *Ang*^ − /−^light strand; and ⑦ *Ang*^ + /+^ light strand) and fold changes in cleavage abundance [③ log2 fold change(KOmean/WTmean) in heavy strand; and ⑤ log2 fold change (KOmean/WTmean]) in light strand] was shown in **b**. Increases are shown in red and decreases in blue. The mitochondrial genome is displayed in the central track ④, with the nucleotide position in base pairs displayed across the exterior; rRNAs are displayed in red, mRNAs in green, tRNAs in blue, and the non-coding region (NCR) in gray. **c** Genome browser view of the 5’- and 3’-end cleavage frequencies of the tRNAs bordering *Rnr1* (*12S*) (left panel) and *Rnr2* (*16S*) (right panel). The cleavage frequencies in *Ang*^ + /+^ and A*ng*^ − /−^ cells at each cleavage site were compared, and relative changes in mean frequency [log2 (KOmean/Ctrlmean)] are also shown. Regions of interest are marked using black boxes

To further validate the cleavage sites bordering *12S* and *16S* rRNA and identify potential additional ANG cleavage sites in mitochondrial RNA transcript, we performed parallel analyses of RNA ends (PARE)-sequencing to investigate the distribution of 5’- and 3’-end across the entire mitochondrial transcriptome in the *Ang*^ − /−^ cells relative to *Ang*^ + /+^ cells during osteoclast fusion. Identical cleavage sites were identified throughout the mitochondrial genome by comparing our PARE-sequencing data with a previously published dataset from mouse hearts (GSE83471),^28^ indicating conserved mtRNA processing mechanisms across various mammalian tissues. Analyses of all canonical cleavage sites revealed a dramatic reduction in reads mapping to the annotated 3’-end of transcripts derived from the heavy strand but not the light strand of the mitochondrial genome in *Ang*^ − /−^ cells compared with *Ang*^ + /+^ cells (Fig. 7a), suggesting that ANG is critical only for 3’ but not for 5’ tRNA processing. A transcriptome-wide map shows the changes of cleavage sites at both the 5’- and 3’-end tRNA processing sites across the entire mitochondrial transcriptome from the *Ang*^ + /+^ relative to *Ang*^ − /−^ cells (Fig. 7b). The cleavage targeting the 3’-end of tRNA occurred predominantly at tRNA^Phe^ before *Rnr1* (*12S* encoding gene) and tRNA^Val^ before *Rnr2* (*16S* encoding gene) (Fig. 7b, red arrow pointed spots). Further calculation of the reads showed approximately 48% and 28% reductions at the 3’ tRNA cleavage sites that border *12S* and *16S* rRNA, respectively, in the *Ang*^ − /−^ cells compared with the *Ang*^ + /+^ cells (Fig. 7c, and Supplementary Tables 1 and 2). The cleavage targeting the 3’-end of tRNA of other mt-encoded genes is also more or less affected by ANG-deficiency (Supplementary Tables 1 and 2). However, there were almost undetectable reads at the 5’ tRNA cleavage sites that border both rRNAs in either *Ang*^ + /*+*^ or *Ang*^ − /−^ cells. In addition, we observed increased reads of small RNA products ( < 15nt) in *Ang*^ − /−^ cells (8.1%) compared with *Ang*^ + /+^ cells (6.7%) (Supplementary Table 3), indicating an increased degradation of the transcripts, likely due to a high load of unprocessed RNAs upon loss of ANG. Together, our data suggest that ANG is crucial for mt-tRNA 3’-end processing, specifically during high-energy-demanding cell-cell fusion.

### mtRNA cleavage activity of ANG is required for myoblast fusion during muscle repair

**Fig. 8:**
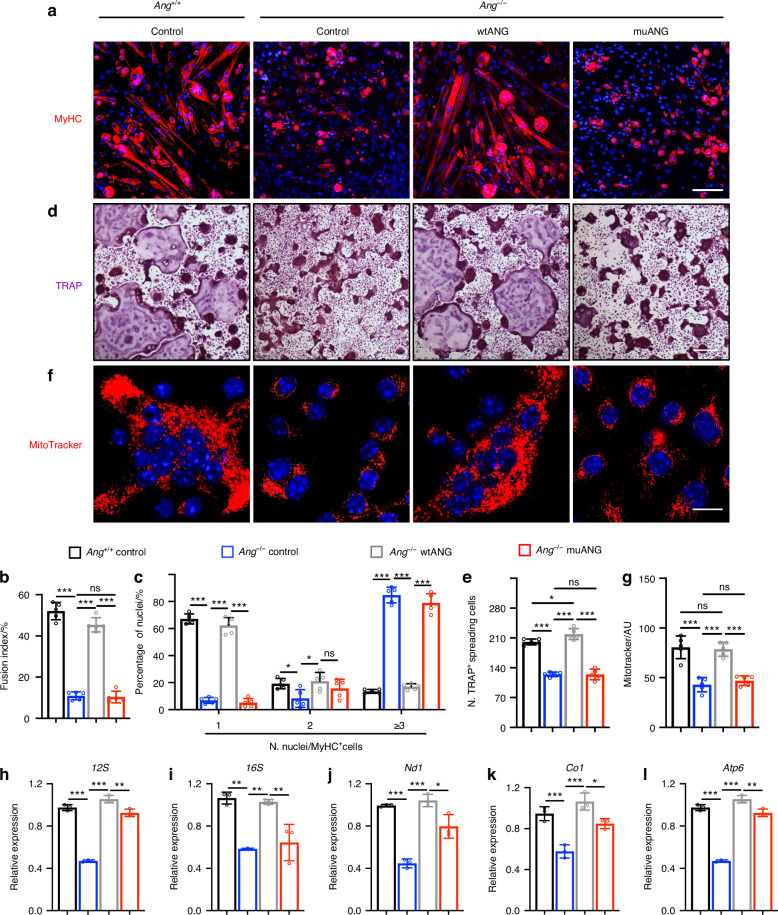
mtRNA cleavage activity of ANG is required for myogenesis and osteoclastogenesis. **a**–**c** Skeletal muscle satellite cells from *Ang*^ + /+^ and *Ang*^ − /−^ mice infected with individual lentiviruses were induced differentiation with 2% horse serum for 7 days to acquire mature myotubes. Representative images of MyHC staining were shown in (**a**). Scale bars, 50 μm. Quantified analyses of the fusion index and the percentages of cells with different numbers of nuclei were shown in (**b**, **c**). *n* = 5 samples per group, and 3 fields per sample were calculated. **d**, **e** Bone marrow Mo/Mac from *Ang*^ + /+^ and *Ang*^ − /−^ mice infected with individual lentiviruses were treated with M-CSF (30 ng/mL) and RANKL (100 ng/mL) for 4 days to acquire mature osteoclasts. Representative images of TRAP staining (**d**) and quantification of the TRAP^+^ spreading cells (**e**). Scale bars, 200 μm. **f**, **g** Bone marrow Mo/Mac from *Ang*^ + /+^ and *Ang*^ − /−^ mice infected with individual lentiviruses were treated with M-CSF (30 ng/mL) and RANKL (100 ng/mL) for 3 days to acquire fusing OCs. Representative images of MitoTracker Red (**f**) and fluorescence intensity quantification (**g**) were shown. Scale bars, 20 μm. **h**–**l** Bone marrow Mo/Mac from *Ang*^ + /+^ and *Ang*^ − /−^ mice infected with individual lentiviruses were treated with M-CSF (30 ng/mL) and RANKL (100 ng/mL) for 3 days to acquire fusing OCs. RT-qPCR analysis of the expressions of mitochondrial rRNAs and mRNAs were shown in (**h**–**l**) *n* = 3. Data are shown as mean ± s.d. and analyzed by one-way ANOVA with Tukey’s multiple comparisons between the indicated groups. ***P < 0.001, **P < 0.01, *P < 0.05, and ns ≥ 0.05

We then evaluated if RNA cleavage activity of ANG is crucial for cell-cell fusion. We constructed lentiviruses carrying either wild-type *Ang* (wtANG) or *K40I-Ang* mutant (muANG) with an HA-tag at the C-terminus (Fig. S16a). The K40I variant has been identified in patients with amyotrophic lateral sclerosis (ALS) and is a loss-of-function mutation known to affect predominantly the ribonucleolytic activity of ANG protein.^58,70,71^ Both wtANG and muANG could be expressed effectively in both mouse satellite cells (Fig. S16b) and Mo/Mac (Fig. S16c) by virus infection. MyHC^+^ muscle fiber formation was significantly impaired in *Ang*^ − /−^ cells compared to *Ang*^ + /+^ cells (Fig. 8a, 2nd vs. 1st image), consistent with Fig. 3f−h. Importantly, forced expression of wtANG restored MyHC^+^ myotubes (Fig. 8a, 3rd vs. 2nd image), significantly increasing the fusion index (Fig. 8b) and the number of nuclei per muscle fiber (Fig. 8c). In contrast, muANG infection failed to achieve the same effect, showing impaired muscle fiber formation similar to *Ang*^ − /−^ cells infected with the empty vector (Control) virus (Fig. 8a 4th vs. 2nd image, and 8b, c). Consistent results were also observed in osteoclast fusion experiments. Virus containing wtANG effectively rescued osteoclastogenesis impaired by *Ang* deficiency, as indicated by the increased number of TRAP^+^ fusing OCs (Fig. 8d, e). However, muANG failed to produce this effect.

We also tested whether wtANG or muANG overexpression affects mitochondrial activities of the fusing cells. While the abundance of mitochondria was dramatically lower in *Ang*^ − /−^ relative to *Ang*^ + /+^ cells (Fig. 8f 2nd vs. 1s image, and 8g), mitochondrial number was restored almost completely in cells infected with wtANG (Fig. 8f 3rd vs. 2nd image, and 8g). However, cells infected with muANG showed no significant difference compared with those infected with the empty vector (Control) in *Ang*^ − /−^ cells (Fig. 8f 4th vs. 2nd image, and 8g), confirming the requirement of the ribonuclease activity of ANG for mitochondrial biogenesis and osteoclast fusion. We then tested whether wtANG or muANG overexpression affects mtRNA processing. *Ang*^ − /−^ cells infected with virus carrying wtANG showed increased mitochondrial rRNAs *12S* and *16S* to the levels of *Ang*^ + /+^ cells (Fig. 8h, i), suggesting full recovery of mitochondrial rRNA processing. The mRNA levels of mitochondrial-encoded genes *Nd1, Co1*, and *Atp6* were all consistently upregulated in wtANG-infected *Ang*^ − /−^ cells (Fig. 8j–l). In contrast, muANG failed to upregulate the expressions of rRNA and any of the mitochondria-encoded genes in *Ang*^ − /−^ cells (Fig. 8h–l).

**Fig. 9:**
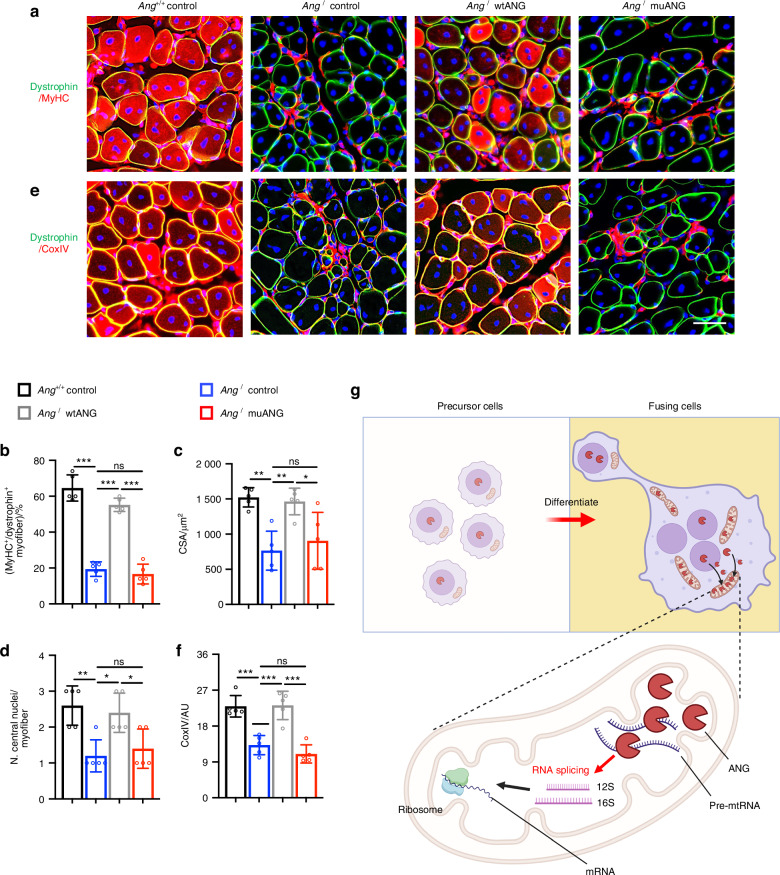
mtRNA cleavage activity of ANG is required for myoblast fusion during muscle regeneration. **a**–**f** Muscle injury was induced by injection of a 1.2% BaCl_2_ solution into GA muscles of four-month-old *Ang*^ + /+^ and *Ang*^ − /−^ mice, followed by administration of the corresponding virus the next day. The GA muscles were harvested for immunostaining analysis after 11 days. Double immunofluorescence staining of the mucle tissue sections with Dysthrophin/MyHC (**a**) and Dysthrophin/CoxIV (**e**) antibodies. Quantified percentage of MyHC⁺ myofibers within Dystrophin-stained sections (**b**), average cross-sectional area (CSA) per myofiber (**c**), average number of central nuclei per myofiber (**d**), and CoxIV fluorescence intensity per mm² of tissue area (**f**) were shown. *n* = 5 samples per group, and 3 fields per sample were calculated. **g** Schema summarizing the main findings in this study. Data are shown as mean ± s.d. and analyzed by one-way ANOVA with Tukey’s multiple comparisons between the indicated groups. ***P < 0.001, **P < 0.01, *P < 0.05, and ns ≥ 0.05

As myoblast fusion is critical for post-injury muscle regeneration,^38^ we examined the effects of ANG on skeletal muscle regeneration following GA muscle injury induced by intramuscular injection of 1.2% BaCl₂. The results showed that the number of MyHC^+^ regenerating myofibers, as well as the average CSA of the myofibers, were greately reduced in the GA muscle of *Ang*^ − /−^ mice compared to *Ang*^ + /+^ mice following injury (Fig. 9a 2nd vs. 1st image, and 9b, c). Intriguingly, local injection of a virus carrying wtANG into the injury sites of *Ang*^ − /−^ mice robustly increased the number of MyHC^+^ regenerating muscle fibers (Fig. 9a 3rd vs. 2nd image, and 9b), whereas virus carrying muANG had minimal effects on myofiber regeneration in *Ang*^ − /−^ mice (Fig. 9a 4th vs. 2nd image, and 9b). Compared to the empty vector-infected group (Control), the wtANG-infected group showed substantial recovery in GA muscle structure, marked by significant increases in muscle fiber size and the number of central nuclei (Fig. 9c, d). In contrast, no significant differences were observed between the muANG-infected and empty vector-infected GA muscles (Fig. 9c, d). To determine whether enhanced muscle regeneration by wtANG expression was attributed to ANG-mediated mitochondrial biogenesis, we analyzed the expression of the mitochondrial marker CoxIV. Consistent with the in vitro findings, while abundant CoxIV^+^ myofibers were present in *Ang*^ + /+^ mice post-injury, CoxIV^+^ myofibers were diminished in *Ang*^ − /−^ mice (Fig. 9e 2nd vs. 1st image, and 9f). However, GA muscles infected with wtANG showed dramatically higher numbers of CoxIV^+^ myofibers compared to the empty vector-infected group (Fig. 9e 3rd vs. 2nd image, and 9f). This restored mitochondrial activation response was not observed in the muANG-infected group (Fig. 9e 4th vs. 2nd image, and 9f). Thus, the RNA cleavage activity of ANG is crucial for mitochondrial biogenesis, which drives cell-cell fusion necessary for muscle regeneration.

## Discussion

Mitoribosome biogenesis, the mitochondrial translation system, is essential for energy production. Defects in this process result in severe, phenotypically diverse diseases, leading to tissue and organ dysfunction, especially during stages of high metabolic demands. Although cell-cell fusion is a highly energy-demanding process, the molecular mechanisms that activate and sustain the mitochondrial translation system during fusion remains largely unknown. In this study, we have identified ANG as a novel mt-tRNA endoribonuclease as a key orchestrator of mitoribosome biogenesis during cell-cell fusion, an energy-intensive process critical for many biological functions in mammals. We demonstrate that in fusing cells, there is a robust translocation of ANG from the nucleus/cytosol to the mitochondria, where ANG directly mediates the cleavage of mt-tRNA bordering rRNAs, driving mitoribosome biogenesis (Fig. 9g). Given that mt-tRNA excision is the critical initial step in the mitochondrial RNA processing and maturation process for producing functional RNAs required for the respiratory chain protein translation, our findings establish ANG as an essential element in the mitochondrial ribosome biogenesis machinery.

We have demonstrated for the first time that ANG is translocated from the nucleus/cytosol to the mitochondria in the differentiating osteoclast and myoblast precursors, especially during cell-cell fusion. Using different approaches, including immunofluorescence staining, western blot, and immune-electron microscopy, we have shown that ANG is expressed at low levels, primarily in the nucleus or cytosol, in the undifferentiated precursors of both lineages of osteoclasts and myoblasts. We observed a greater expression of ANG and its robust translocation to mitochondria in the fusing osteoclasts and myoblasts. Interestingly, we noted that ANG is localized primarily in the mitochondrial matrix and inner membrane, where mitoribosome biogenesis occurs. It remains unclear how ANG is translocated into mitochondria during cell-cell fusion. By using an iMLP algorithm,^62^ we found that a putative internal mitochondrial matrix targeting-like sequence (iMTS-Ls), which has β-hairpin elements targeted to mitochondria,^72^ is located between amino acids 17 to 99 of human ANG, and between amino acids 14 to 96 of mouse ANG. The mitochondrial chaperones may also facilitate ANG transport to mitochondria. Future work into detailed mitochondrial targeting mechanisms would help in a better understanding of its function in mitoribosome biogenesis during the process of cell-cell fusion. Most previous studies have focused on the nuclear localization of ANG in different types of cells. ANG with a nuclear localization signal drives the accumulation of ANG in the nucleolus, where it stimulates rRNA transcription essential for ribosome biogenesis of the cells.^73,74^ Given that eukaryotic cells harbor 2 interdependent genomes encoded by the nucleus and mitochondria that need to communicate constantly to balance energy needs,^75–77^ our results suggest that ANG may be a critical mediator in the communication between mitochondria and the central nuclear program as it localizes in both organelles.

Perhaps the most striking finding of this study is the identification of the requirement of ANG for 3’ mt-tRNA processing. Upon *Ang* deletion, there was an accumulation of the unprocessed tRNA^Phe^-*12S* rRNA and tRNA^Val^-*16S* rRNA specifically in fusing cells. Moreover, the increase in the mRNA expression of *12S*- and *16S*-encoding genes was completely abolished in *Ang*-deficient cells. Of note, neither the levels of unprocessed rRNA precursors nor the levels of mature ribosomal mRNA expression were affected by ANG deficiency in the undifferentiating precursors without cell-cell fusion. Therefore, a robust mt-tRNA processing is activated specifically during cell-cell fusion, and ANG appears to be the major player only in fusing cells but not in undifferentiated precursor cells. Supporting this assumption, both 5’RACE and PARE-seq results demonstrated dramatically reduced cleavage at the 3’-end but not the 5’-end of the mt-tRNAs bordering the rRNAs and almost all of the mt-encoded RNAs in the absence of *Ang* only in fusing cells. As a result, the levels of both mitochondrial DNA transcript expression and protein expression of mitochondria-encoded genes were diminished with impaired mitochondrial respiration function in ANG-deficient cells. Therefore, *Ang* deficiency traps the mitochondrial RNAs in precursor transcripts and thereby leads to profound reduction of mature RNAs and impaired respiratory chain protein translation. The finding that a virus containing wild-type ANG, but not the mutant ANG with disrupted ribonucleolytic activity, effectively rescued cell-cell fusion impaired by *Ang* deficiency firmly implies that mtRNA cleavage activity of ANG is essential for this fundamental cellular process.

We present an important new finding from the PARE-seq and 5’RACE analyses. In other mammalian cells, the 5’-leader pre-RNA cleavage normally occurs prior to 3’-trailer processing.^26,28^ However, the 3’-end of tRNA^Val^ and tRNA^Phe^ are cleaved more frequently than their 5’-end in the fusing cells, suggesting that some ribosomal proteins may be able to assemble in the absence of 5’-end processing of *12S* and *16S* rRNA. It is interesting to note that although the cleavage of the 5’-end of tRNAs bordering rRNAs was not reduced in *Ang*-deficient cells relative to WT cells in 5’RACE assays, qRT-PCR of the 5’-end of unprocessed *12S*-tRNA^Phe^ and *16S*-tRNA^Leu^ products still accumulated more in *Ang*^ − /−^ than in *Ang*^ + /+^ cells. The reason for this discrepancy may be that the impaired processing of the 3’-end causes markedly increased levels of pre-RNAs, including newly transcribed and unprocessed RNAs, leading to an increase in 5’-end RNA products. It has been demonstrated that *ELAC2* (RNase Z encoding gene) is essential for the 3’ mitochondrial tRNA processing enzyme, and its activity is nonredundant in heart and skeletal muscles.^30,36^ Our observation suggests that while RNase Z is responsible for 3’ mt-tRNA processing in undifferentiated precursor cells at baseline, ANG translocated into mitochondria may become a predominant endoribonuclease in mt-tRNA excision during cell fusion. Indeed, our 5’RACE analysis using *Elac2* knockdown and *Ang*^ − /−^ cells suggests that ANG is able to achieve 3’-end tRNA cleavage more efficiently than RNase Z during osteoclastogenesis. Nevertheless, the possibility that ANG may also facilitate the processing efficacy of RNase Z needs to be further confirmed.

Our in vivo results demonstrate that ANG-mediated mitochondrial ribosome biogenesis contributes to osteoclastogenesis during postnatal bone growth and adult bone remodeling. Although osteoclasts have long been recognized to be rich in mitochondria,^78,79^ the way in which mitochondria biogenesis is activated during osteoclastogenesis is much less clear. Increased mitochondrial biogenesis during osteoclast formation and function was shown to be regulated by PGC-1β and iron uptake by transferrin receptor 1 and its subsequent delivery to mitochondria.^18,80–82^ Our study reveals that during osteoclast fusion, there is not only an increase in the number of mitochondria, but that they undergo a dramatic 5-fold increase in average size. Moreover, the activation of ribosomal biogenesis in the nucleus occurs during the early stage of osteoclast differentiation, before cell-cell fusion has started, whereas mitochondria ribosomal biogenesis becomes predominant when osteoclast fusion begins. ANG loss leads to severe morphological changes of the mitochondria, as well as mitochondrial dysfunction of osteoclast precursors, leading to abolished osteoclast fusion and failure of osteoclastogenesis. As a result, *Ang*^ − /−^ mice exhibit an abnormally higher bone mass phenotype in both growing young mice and adult mice compared with their WT littermates. We previously demonstrated that osteoclast-derived ANG supports type H vessels in the metaphysis, thereby promoting angiogenesis–osteogenesis coupling.^68^ In *Ang*^ − /−^ mice, we observed a reduction in metaphyseal type H vessels accompanied by decreased CoxIV expression compared with WT mice, indicating impaired mitochondrial activity in endothelial cells and suppressed vessel formation, which likely leads to reduced osteogenesis and bone formation in this region. However, ANG-deficient mice paradoxically exhibit increased bone mass relative to WT mice. This discrepancy reflects distinct and competing biological processes affected in this global ANG knockout model. Specifically, ANG deficiency causes a profound reduction in osteoclast number and activity throughout the skeleton, resulting in markedly suppressed bone resorption. As a result, although bone formation is indirectly decreased in the metaphysis due to impaired angiogenesis-coupled osteogenesis, the dominant and direct suppression of bone resorption ultimately outweighs this effect, leading to a net increase in bone mass. We demonstrate that ANG is important for myogenesis during skeletal muscle homeostasis. Histological analysis of skeletal muscles from 4-month-old *Ang*^ − /−^ mice revealed reduced muscle fiber cross-sectional area and fewer myonuclei in myofibers, which failed to form large myotubes. Functionally, *Ang*^ − /−^ mice exhibited significant defects in muscle performance and endurance, as assessed by treadmill exhaustion test, highlighting ANG’s critical role in maintaining muscle homeostasis. We further demonstrate that mtRNA cleavage activity of ANG is crucial for tissue repair and regeneration following injury. ANG deficiency impaired muscle regeneration after injury and led to abnormal bone healing following bone injury. Moreover, skeletal muscle regeneration was significantly improved by local injection of a virus containing wild-type ANG, but not by the mutant ANG lacking cleavage activity. Notably, the K40I mutation in ANG, identified in patients with amyotrophic lateral sclerosis (ALS),^58,70,83^ disrupts ANG cleavage activity. Although ALS is primarily regarded as a neurodegenerative disorder affecting upper and lower motor neurons, our findings suggest that loss of ANG-mediated mtRNA cleavage in skeletal muscle may also contribute to disease pathogenesis. ANG belongs to the RNase A family, whose members are broadly conserved across vertebrate evolution. *ANG* gene has been identified in fishes, reptiles, birds, and mammals, with ANG-like sequences present even in basal vertebrates such as teleosts,^84^ suggesting that the core RNase/ANG module emerged early in vertebrate evolution and has been maintained in diverse species. The conservation of RNase catalytic residues and key structural features further supports functional preservation despite lineage-specific diversification.^85^ Although humans possess a single *ANG* gene, the mouse genome has undergone lineage-specific expansion of the *Ang* locus, resulting in at least five functional paralogues (*Ang1*, *Ang2*, *Ang4*, *Ang5*, and *Ang6*) as well as multiple pseudogenes.^86^ Among these, mouse *Ang1* (*mAng1*) shares the closest sequence and structural similarity with human *ANG* (*hANG*), retaining the conserved catalytic features and overall fold characteristic of ANG and exhibits robust angiogenic activity comparable to the human protein.^87,88^ In contrast, the other murine *Ang* paralogues show greater sequence divergency and functional specialization. Consistently, only mAng1 closely mirrors the functional properties of hANG. In particular, *Ang1*-deficient mouse models, including the *Ang*^ − /−^ mice used in the present study, have been shown to recapitulate key aspects of human pathology across diverse tissues and cell types.^89–91^ In this study, we found that both osteoclast and skeletal muscle lineages derived from *Ang*^ − /−^ mice exhibit pronounced defects in mitochondrial biogenesis and cell–cell fusion, which were rescued by re-expression of wile-type *mAng1* but not by catalytically inactive mutant *mAng1*. These results demonstrate an indispensable role for the ribonuclease activity of mAng1 in regulating mitochondrial biogenesis and fusion-related cellular behaviors and argue against functional compensation by other *mAng* paralogs in these contexts. However, we noticed that *Ang*^ − /−^ mice were born at Mendelian ratios and did not display overt developmental abnormalities, suggesting that compensatory mechanisms may buffer the loss of *mAng1* in vivo during embryonic myogenesis. Such compensation could reflect partial functional redundancy among murine ANG paralogues in a subset of developing muscle fibers and/or developmental robustness that allows myogenesis to proceed via alternative pathways. Future studies will be required to determine whether other *mAng* genes are upregulated in *Ang1*-deficient muscle cells during embryonic development and whether they contribute functionally to myocyte fusion.

ANG has long been recognized as a pleiotropic factor involved in angiogenesis, neuroprotection, and cellular stress responses. Our finding that ANG regulates mitochondrial RNA processing to promote mitoribosome biogenesis suggests a potential unifying mechanism underlying this functional diversity. Because mitochondrial translation and mitoribosome biogenesis are energetically demanding processes, particularly in tissues with high metabolic requirement, ANG-mediated regulation of mitochondrial gene expression may facilitate rapid metabolic remodeling during angiogenesis, neurite outgrowth, tissue regeneration, and cellular stress adaptation. If this model is correct, impaired ANG-dependent mitochondrial regulation could contribute to defective tissue repair/regeneration and increased vulnerability to neurodegenerative and stress-related conditions, especially in aging or disease contexts characterized by mitochondrial dysfunction. In the nervous system, multiple studies have demonstrated that ANG promotes neuronal survival and neurite outgrowth,^92,93^ whereas ALS-associated ANG variants frequently lose these neuroprotective activities,^94^ consistent with a loss-of-function mechanism. Conversely, in certain pathological contexts such as tumor angiogenesis, inhibition of ANG-dependent mitoribosomal biogenesis may represent a potential therapeutic strategy. Future studies will be needed to define the context-dependent roles of ANG-mediated mitochondrial regulation in physiology and disease.

Importantly, a previous study demonstrated that chronic systemic recombinant human ANG (hANG) (1 mg, i.p., three times per week for multiple weeks) in an ALS animal model improved motor neuron function and prolonged survival,^95^ supporting the feasibility and potential therapeutic benefit of extended systemic dosing in a disease context. Nevertheless, for both preclinical and clinical translation, it will be essential to establish optimal dosing parameters, conduct longer-term follow-up studies, and systematically evaluate angiogenic and oxidative-stress-related effects. Consideration of tissue-restricted or inducible delivery strategies may also help mitigate potential risks associated with chronic exposure.

## Materials and methods

### Mice

*Ang*^ − /−^ mice were generated by Dr. Guo-Fu Hu’s laboratory from the Molecular Oncology Research Institute, Tufts Medical Center.^91^ Briefly, *Ang*^ − /−^mice were generated by crossing *Ang1* gene floxed mice with EIIa-Cre mice and were backcrossed eight generations to obtain *Ang*^ − /−^ mice in pure C57BL/6 background. Unless otherwise stated, age-matched 1-, 2- or 4-month-old gender-matched and wild-type littermates were used. The genotypes of the *Ang*^ − /−^ mice were determined by PCR analyses of genomic DNA extracted from mouse-tail snips using the following primers: F12 forward, 5′-AGG GTG GAA CTT CAG GAT TCA AG-3′ and DL-1 reverse, 5′-TCT TGA TCC TAA ACT CCT TTC CAA AG-3′. Mice were housed at 25°C in a 12-hour light cycle with food and water ad libitum. All animals were maintained in the animal facility of The Johns Hopkins University School of Medicine under protocol MO24M35 approved by the Institutional Animal Care and Use Committee of The Johns Hopkins University, Baltimore, MD. All protocols and procedures followed the guidelines of the Institutional Animal Care and Use Committee of The Johns Hopkins University, Baltimore, MD.

### In vitro osteoclastogenesis induction and measures

In vitro osteoclastogenesis assays were performed as described previously.^68,96^ Briefly, mouse bone marrow cells were harvested from 4-week-old male mice and cultured with alpha minimum essential medium (α-MEM) containing 15% fetal bovine serum (FBS), 100 U/mL streptomycin sulfate (Sigma-Aldrich) and 100 U/mL penicillin (Sigma-Aldrich) in 10-cm culture dishes at 37 °C in 5% CO_2_ humidified incubator for 24 h. Cells were then cultured with 30 ng/mL M-CSF (Amizona Scientific, AM10003) for adhesion, and 100 ng/mL RANKL (Amizona Scientific, AM10004) to induce osteoclast differentiation, yielding pre-fusing osteoclast precursors after 2 days and fusing osteoclasts after 3 days. The culture medium was changed every second day in all experiments. Osteoclast formation was detected by TRAP staining using commercially available kit (Sigma, 387 A). For actin ring staining, cells were cultured on glass coverslips in the presence of M-CSF and RANKL for 4 days, after which cells were fixed in 4% paraformaldehyde, permeabilized in 0.1% Triton X-100, rinsed in PBS, and immunostained with rhodamine-phalloidin (Invitrogen, A34055, 1:1 000). For bone resorption pit assays, suspension Mo/Mac were seeded on bone slices (IDS, DT-1BON1000-96) and induced osteoclast formation with 30 ng/mL M-CSF and 100 ng/mL RANKL for 10 days. Bone slices were washed with 6% sodium hypochlorite and PBS and observed on microscope.

### In vitro myogenesis induction and measurements

Primary satellite cells were derived from the hindlimb muscles of 2-month-old male mice. Briefly, the hindlimb muscles were isolated and minced mechanically, followed by enzymatic digestion at 37 °C for 1 h using 400 IU/mL collagenase II (Sigma-Aldrich, C2-BIOC). The digested slurry was sequentially passed through 70 μm and then 30 µm cell filters. After a pre-plating step to remove contaminant cells, satellite cells were cultured on 10% Matrigel-coated dishes (Corning, 354234) in growth medium (Ham’s F-10 medium with 20% FBS, supplemented with 10 ng/mL basic fibroblast growth factor). To induce differentiation, the cells were incubated in DMEM (Gibco, 12491015) supplemented with 2% donor horse serum (Gibco, 26050088) which refreshed daily. The fusion index was derived by calculating the proportion of nuclei within MyHC^+^ myotubes (contained ≥2 nuclei) relative to the total number of nuclei analyzed. For nuclear quantification, the percentages of MyHC^+^ cells containing 1, 2, and ≥3 nuclei were caculated relative to the total MyHC^+^ cell population.

### Small interfering RNAs and viral infection

The transient inactivation of *Elac2* was achieved using MISSION® Predesigned siRNAs obtained from Sigma-Aldrich and transfected into Mo/Mac (50% confluent) using Lipofectamine RNAiMAX (Invitrogen, 13778-150) according to the manufacturer’s protocol. A nontargeting scrambled (Scr) siRNA sequence (Sigma-Aldrich, SIC001) was used for control siRNA. Impedance measurements were performed 72 h after transfection. For viral infection, cells were infected with individual lentiviral constructs carrying wild-type *Ang* (LV-EF1a-mAng-C-HA-WPRE), *Ang* with point mutation K40I (LV-EF1a-Ang K40I-C-HA-WPRE), or an empty vector control (LV-EF1a-HA-WPRE) with HA-tag. The transfection efficiency was confirmed by western blot at 72 h after infection.

### Nuclear and mitochondrial isolation from the cells

Nuclear of cultured cells were extracted by nuclear extraction kit (Abcam, ab113474) according to the manufacturer’s instructions. Mitochondrial isolation was performed as previously described.^97,98^ Briefly, cells were washed with cold PBS and collected to 5 mL glass homogenizer with MitoPrep buffer (0.225 mol/L mannitol, 0.075 mol/L sucrose, 20 mmol/L HEPES, pH 7.4). After 30 Dounce strokes, homogenate was centrifuged at 900 × *g* for 10 min at 4 °C. Supernatant was saved, and the pellet resuspended with MitoPrep buffer was subjected to extra Dounce strokes. After centrifugation, all the collected supernatant was centrifuged at 12 000 x *g* for 15 min at 4 °C to get the mitochondrial pellets. The final pellet was resuspended in protein or RNA lysis buffer for downstream analyses.

### Quantitative RT-PCR analyses

RNAs were extracted from cells or mitochondria using RNeasy Mini Kit (QIAGEN, 74014) according to the manufacturer’s instructions. Reverse transcription was performed using SuperScript First-Strand Synthesis System (Invitrogen, 11904018). Reverse transcription products were analyzed with SYBR GreenMaster Mix (QIAGEN, 330603) in QuantStudio 3 (ThermoFisher Scientific). Target-gene expression was normalized to total cell’s β-Actin messenger RNA and calculated with the 2-ΔΔCt method. Primer information is listed in Supplementary Table 4.

### RNA library construction and PARE-Seq

Mitochodrial RNAs was extracted from mitochondria according to the protocol provided in the RNeasy Mini Kit (QIAGEN, 74014). PARE libraries were prepared as described previously.^99^ Briefly, the cleaved mitochondrial polyA-RNA samples were ligated to a single-stranded 5′ RNA adaptor using RNA ligase, followed by reverse transcription and amplification. The PCR products were digested with MmeI, a type IIS restriction enzyme, to generate equal-sized fragments, which were recovered by PAGE. After ligation with a 3′ adapter containing degenerate nucleotides in the overhang region, the digested RNAs were amplified and gel-purified for deep sequencing on the Illumina NovaSeq X Plus by CD Genomics (Shirley, USA). To analyze the sequencing data, sequencing reads were initially aligned to the mouse genome (GRCm39) with Bowtie2 (v2.5.3) using default parameters and returning only unique aligning reads. The comparison results were converted into bed format by samtools and bedtools, and the positive and negative strand-specific depth of read coverage at the 5’-end position of aligned reads was calculated by python. The read depth was normalized to reads per million and converted to bedGraph format by bedtools (v2.31.1) for visualization on the Integrative Genomics Viewer (IGV). Relative fold changes were calculated as log_2_ fold changes of the mean normalized 5’-end coverage for *Ang*^ − /−^ to *Ang*^ + /+^, plus a pseudo count of one. The sequence logo plot based on the normalized read depth and relative fold change was produced by tbtools (v2.070).

### 5’RACE analysis

Mitochondria RNA was used for 5’RACE assays using 5’RACE System for Rapid Amplification of cDNA Ends (Invitrogen, 18374058) according to the manufacturer’s instructions. Briefly, the GSP primer was diluted and mixed with RNA, which was then denatured at 70 °C and cooled on ice, followed by the addition of SuperScript™ II RT for reverse transcription. After RNase treatment, cDNA was purified with SNAP columns and tailed with dCTP using TdT enzyme. Nested PCR was used to amplify and purify the target fragment. The amplified product was analyzed by 2.5% agarose gel electrophoresis and DNA-sequencing.

### Basic processing and gene ontology (GO) analysis of different cell clusters in publicly available scRNA-Seq datasets

The cultured osteoclasts scRNA-seq dataset (GSE147174) was obtained from an open-access platform (https://www.ncbi.nlm.nih.gov/sra) and contained single-cell transcriptome profiling of mouse osteoclast precursors at different differentiation stages.^53^ The Cell Ranger (v7.1.0) count pipeline aligned sequencing reads from raw FASTQ files to the Mus musculus genome reference sequence (GRCm39), counted the reads, and excluded barcodes corresponding to background noise to generate a Market Exchange Format (MEX) expression matrix. Count matrices were initialized by the R package Seurat, integrated, normalized and corrected by the R package Harmony. Cells expressing fewer than 200 genes or more than 5% mitochondrial reads in all genes were removed. Cells that passed quality-control criteria comprise 1 872, 1 759, and 1 208 cells from 0-day, 1-day, and 3-day, respectively. The ElbowPlot function was used to determine the first 19 significant principal components for Uniform Manifold Approximation and Projection (UMAP) to dimensionality reduction to 13 cell clusters, which were aggregated and annotated into 9 cell types according to markers provided in the original work. Pseudotime trajectories of osteoclasts differentiation were analyzed using Slingshot (v2.10.0) and monocle3 (v1.4.26). Single-cell RNA-seq data filtered for Mo/Mac, early OCPs, pre-fusing OCPs and fusing OCs were processed in UMAP space. Trajectories were inferred starting from Mo/Mac and visualized with cell types color-coded. The expression of mitochondrial encoded genes were shown in jittered scatter plots. A heatmap illustrating the expression patterns of osteoclast differentiation marker genes across different cell types was generated. Gene Ontology (GO) terms were utilized to demonstrate functional enrichment in various cell types.

For the analysis of muscle differentiation, a similar investigation was performed on the single-cell RNA-seq dataset of homeostatic skeletal muscle satellite cells induced differentiation (GSE126834).^54^ Cells expressing fewer than 200 genes, more than 2.5% mitochondrial reads in all genes were removed. Cells that passed quality-control criteria comprise 2 226 and 4 344 cells from homeostatic skeletal muscle satellite cells and differentiated myoblasts. The filtered data were normalized and subjected to Principal Component Analysis (PCA), with batch effects across different samples corrected using the Harmony method. Thirteen principal components were selected following two specific criteria: the cumulative variance explained exceeded 90%, with each principal component contributing less than 5%, or the variance change between consecutive components was greater than 0.1%. The smaller number from these two approaches was chosen as the final number of principal components. Dimensionality reduction was performed using UMAP, followed by manual annotation, resulting in the identification of five major cell types. Muscle cell differentiation pseudotime trajectories were analyzed with Slingshot. Single-cell RNA-seq data filtered for satellite cells, myoblasts, fusing myocytes, and myotubes were mapped in UMAP space. Trajectories were inferred from satellite cells and visualized with color-coded cell types. The expression of marker genes and *Ang* across various cell types were displayed using a stacked violin plot and a heatmap, respectively. Differential gene expression analysis was conducted for the four muscle lineage cell types, for Gene Ontology (GO) enrichment analysis and Kyoto Encyclopedia of Genes and Genomes (KEGG) enrichment analysis.

### Seahorse respiration phenotype measurements

Seahorse respiration phenotype measurements were performed as previously described.^100,101^ Briefly, Mo/Mac were seeded in a Seahorse 96-well cell culture microplate at a density of 5 × 10^4^ cells/well and induced for osteoclastogenesis with 30 ng/mL M-CSF and 100 ng/mL RANKL. The cells were equilibrated for 1 h at 37 °C in a CO_2_-free incubator, followed by measured using a Seahorse XFe96 Extracellular Flux Analyzer. Testing of mitochondrial function was initiated by three baseline OCR measurement cycles. These were followed by the sequential injection of oligomycin (2 μmol/L final concentration), FCCP (2 μmol/L), and a mixture of rotenone (1 μmol/L) and antimycin A (1 μmol/L). OCR was monitored before the first injection and upon each injection with 3 cycles of 1 min-mixing followed by 3 min-measurement at 37 °C.

### Transmission electron microscopy for detection of mitochondrial morphologic and structural changes

Cultured cells were fixed with 4% paraformaldehyde and 1% glutaraldehyde at 4 °C overnight, rinsed in buffer containing 3% sucrose, and osmicated in 1.5% potassium ferrocyanide-reduced 1% osmium tetroxide with 5 mmol/L magnesium chloride for 2 h. The cells were then rinsed in 100 mmol/L maleate buffer with 3% sucrose, en bloc stained with 2% uranyl acetate for 1 h, and dehydrated at 4 °C in a series of ethanol concentrations up to 70%, followed by 100% ethanol at room temperature. Eponate 12 was used for embedding after a propylene oxide transition. Ultra-thin sections (80 nm) were collected on formvar-coated 200-mesh nickel grids and contrasted with 2% uranyl acetate for transmission electron microscopy (Hitachi 7600 TEM). The number and size of mitochondria, as well as the width of the cristae, were determined in ImageJ. The abundance and morphology of cristae were evaluated according to the previously described.^102^

### Immunoelectron microscopy for detection of ANG in mitochondria

The 80 nm-thick ultra-thin sections were exposed to 3% sodium periodate solution for 30 min, subsequently heated in 10 mmol/L citrate buffer (pH 6.2) at 95 °C for 20 min to retrieve antigens. The specimens were blocked in TBST buffer supplemented with 1% goat serum and 1% BSA, followed by overnight incubation at 4 °C in the blocking solution containing the anti-ANG antibody (generated by Dr. Guo-Fu Hu’s laboratory, 1:20).^88,89^ Afterward, the sections were incubated for 2 h at room temperature with 6 nm gold-conjugated secondary antibodies (Jackson ImmunoResearch, 115-195-146, 1:40). After rinsing with distilled water, the grids were fixed in 2% glutaraldehyde with 100 mmol/L cacodylate buffer for 5 min and contrasted using 2% uranyl acetate for immunoelectron microscopy. For quantitative analysis, the number of gold-labeled particles per mitochondrion was counted.

### Cellular immunofluorescence and MitoTracker staining

Cells were cultured in chamber slides for cellular immunofluorescence. Fresh cells were fixed with cold methanol and permeabilized with 0.05% triton X-100. After washing, cells were blocked with 5% goat serum for 1 h at 37 °C and overnight at 4 °C with anti-ANG (generated by Dr. Guo-Fu Hu’s laboratory, 1:200), anti- CoxIV (Cell Signaling Technology, #11967, 1:200), anti-AIF (Cell Signaling Technology, #5318, 1:200), and anti-MyHC(Invitrogen, 14-6503-95), following at 37°C with fluorescent secondary antibodies (Cell Signaling Technology, #4412 & #8889, 1:200) or Phalloidin (Invitrogen, A34055, 1:1 000). Cells were performed nuclei staining by DAPI and fluorescence imaging on Zeiss 880 Airyscan. For MitoTracker staining, cultured cells were incubated with MitoTracker Red CMXRos (Invitrogen, M7512) at 37 °C for 15 min in accordance with the manufacturer’s instructions. Imaging of MitoTracker Red fluorescence was performed on Zeiss 880 Airyscan.

### Mito-FUNCAT assay (mitochondrial fluorescence non-canonical amino acid tagging)

Mitochondrial protein synthesis was evaluated using an HPG incorporation–based Mito-FUNCAT assay combined with click chemistry according to the manufacturer’s instructions (Invitrogen, C10429). Briefly, cells subjected to RANKL stimulation were cultured in methionine-free DMEM supplemented with 100 μmol/L Click-iT® HPG for 2 h at 37 °C, with 100 μg/mL cycloheximide included to selectively inhibit cytosolic translation. Cells were rinsed with PBS and fixed in 4% formaldehyde for 15 min at room temperature, followed by permeabilization with 0.5% Triton X-100 for 20 min. Newly synthesized proteins were fluorescently tagged via exposure to the Click-iT® Plus OPP reaction cocktail for 1 h in the dark. To visualize the mitochondrial network, cells were blocked with 1% BSA and probed with an anti-CoxIV antibody overnight at 4 °C, then incubated with the appropriate secondary antibodies for 1 h at 37 °C. Nuclei were counterstained with 1× HCS NuclearMask™ Blue solution for 30 min at room temperature prior to fluorescence imaging.

### Protein extraction and western blot analysis

Cells and cell component were lysed in RIPA lysis buffer and diluted with loading buffer. 15 ug protein was separated by SDS-PAGE and electrotransferred to a polyvinylidene difluoride (PVDF) membrane. subsequently, the membrane was blocking for 1 h at 37 °C and incubated with anti-ANG (generated by Dr. Guo-Fu Hu’s laboratory, 1:1 000), anti-CoxII (Cell Signaling Technology, #31219, 1:1 000), anti-Lamin B1 (Santacruz, sc-374015, 1:200), anti-NDUFB6 (Abcam, ab110244, 1:2 000), anti-UQCRC2 (Abcam, ab14745, 1:2 000), anti-SDHA (Cell Signaling Technology, #5839, 1:1 000), anti-CoxI (Cell Signaling Technology, #62101, 1:1 000), anti-ATP5A1 (Cell Signaling Technology, #18023, 1:1 000), anti-HA-Tag (Cell Signaling Technology, #3724, 1:1 000), β-actin (Proteintech, 81115-1-RR, 1:5 000), anti-NFATc1 (Santacruz, sc-7294, 1:200), and anti-CTSK (Santacruz, sc-48353, 1:200) overnight followed by secondary antibodies for 1 h at 37 °C. Proteins were visualized with chemiluminescence detection reagents followed by autoradiography.

### In vivo osteoclastogenesis and bone phenotype analyses

Mice of 1- or 4-month-old were sacrificed and perfused with PBS. Femora were dissected and fixed overnight in 10% formalin at 4 °C. The bone tissue was scanned and analyzed by high-resolution μ-CT scanner (Skyscan 1172, Bruker MicroCT) with a voltage of 65 kV, a current of 153 mA, and a reslution of 9 μm per pixel. For femur of 1-month-old mice, the region of interest for the primary spongiosa was selected within 1 mm below the distal epiphyseal growth plate. For femur of 4-month-old mice, the region of interest for the primary spongiosa was selected within 2 mm below the distal epiphyseal growth plate. The 3D structural parameters included: bone mineral density (BMD), relative bone volume or bone volume fraction (BV/TV), trabecular number (Tb.N), and trabecular thickness (Tb.Th). For TRAP staining of bone tissue sections, decalcified bone tissues were embedded in paraffin, and 4 μm–thick longitudinally oriented sections of bone were collected for TRAP staining using commercially available kit (Sigma, 387 A), followed by counterstain with methyl green. To assess the callus area, bone sections from drill-hole injury models were stained with hematoxylin solution (Sigma-Aldrich, MHS32) and eosin-Y (Epredia, 7111). For immunofluorescence staining, decalcified bone tissues were embedded in OCT, and 13 μm–thick longitudinally oriented sections of bone were used for immunofluorescence staining. Briefly, after washing, permeabilizing, and non-specific site closure, the frozen sections were stained with anti-VPP3 (Abcam, ab73404, 1:200), anti-CoxIV (Cell Signaling Technology, #11967, 1:200), anti-CD31 (R&D Systems, FAB3628G, 1:50), anti-Emcn (Santa Cruz, SC-65495, 1:50) primary antibodies at 4 °C overnight. Samples were then incubated with Alexa Fluor® 488, 594, or 647-conjugated secondary antibodies (Invitrogen, 1:200) at 37 °C for 1 h. Cell nuclei were re-stained with DAPI and observed on an upright fluorescence microscope (Olympus, BX53) or Zeiss 880 Airyscan. The intensity profiles and the number of colocalized cells per bone surface were acquired with ImageJ software.

### Drill-hole injury model

Ten-week-old male mice were used to establish a femoral drill-hole injury model. Anesthesia was induced with 1.5% isoflurane in medical air (78% N₂ and 21% O₂) for 15 min and maintained at 1.25% isoflurane throughout the surgical procedure. Under anesthesia, the periosteum at the mid-diaphysis of the anteromedial femur was gently scratched longitudinally using a needle tip. A standardized circular cortical bone defect (0.8 mm in diameter) was subsequently generated at the mid-diaphysis of the femur using a micro-drill. After defect creation, bone debris was carefully removed, and the muscle and skin layers were closed with absorbable sutures. Femora were harvested 14 days post-injury for subsequent analyses.

### In vivo myogenesis and skeletal muscle phenotype analyses

The GA muscles were harvested from 4-month-old mice treated with perfusion, and fixed overnight in 10% formalin at 4 °C. For the evaluation of tissue morphology, 4 µm-thick transverse sections of paraffin-embedded GA muscles were prepared, following previously established protocols.^103^ Hematoxylin and eosin (H&E)-stained sections were examined, and myofiber cross-sectional area (CSA) was quantified. For immunofluorescence staining, 13 μm–thick frozen sections of GA muscles were incubated with anti-CoxIV (Cell Signaling Technology, #11967, 1:200) and anti-Dystrophin (Abcam, ab15277, 1:200) primary antibodies. For quantitative analysis, the CSA, intensity profiles, and the number of nuclei per myofiber were acquired with ImageJ software.

### Skeletal muscle injury and in vivo myoblast fusion assay

At 14 weeks of age, male mice received intramuscular injections of 20 µL of 1.2% BaCl₂ (Sigma-Aldrich) in saline into the GA muscle to induce necrotic injury, as described previously.^40^ 10⁷ TU of lentivirus was administered to the GA muscles 24 h after injury. To assess myoblast fusion in vivo, the GA muscles were cryosectioned 11 days post-lentivirus injection. For fusion evaluation, the percentage of eMyHC^+^ myofibers within dystrophin-stained regions, the average CSA, and the number of central nuclei per myofiber were quantified. To assess mitochondria biogenesis, the intensity profiles of CoxIV were determined in ImageJ.

### Training and fatigue experiments

After three consecutive days of acclimatization on a motorized treadmill at a speed of 10 m/min for 30 min each day, mice were subjected to a run-to-exhaustion protocol.^104,105^ The protocol involved a gradual increase in speed from 0 to 20 m/min on a 10% grade, while the speed was increased by 2 m/min every 5 min until reaching 20 m/min, followed by an exhaustion run at a constant speed of 20 m/min until the mice reached exhaustion. Exhaustion was determined when the mouse remained on the electric shock grid at the rear of the treadmill for more than 5 s. The workload was calculated using the formula: Workload = body weight (kg) × 9.8 (J/kg × m) × vertical running meters (m).

### Statistical analysis

All images resulted from at least three independent experiments with similar results. All data were expressed as mean ± s.d. using GraphPad Prism 10.0.0 (GraphPad Software, San Diego, CA, USA). One-way ANOVA followed by Tukey’s *t* tests, two-way ANOVA followed by Tukey’s *t* tests or unpaired Student’s *t* tests were used to compare the means among groups. Significance levels were set at *P* < 0.05 and indicated by “*“, *P* < 0.01 by “**“, and *P* < 0.001 by “***“; ns not significant.

## Supplementary information

Supplementary Figure S1

Supplementary Figure S2

Supplementary Figure S3

Supplementary Figure S4

Supplementary Figure S5

Supplementary Figure S6

Supplementary Figure S7

Supplementary Figure S8

Supplementary Figure S9

Supplementary Figure S10

Supplementary Figure S11

Supplementary Figure S12

Supplementary Figure S13

Supplementary Figure S14

Supplementary Figure S15

Supplementary Figure S16

Supplementary Table S1

Supplementary Table S2

Supplementary information

## Data Availability

The raw PARE-Seq datasets have been deposited in the NCBI Sequence Read Archive (SRA) under accession PRJNA1453620 and will be accessible upon manuscript acceptance.
