# Supplementary figures and images for: Angiogenin mediates cell-cell fusion as a mitochondrial RNA processing enzyme
Source: Bone Res. 2026 Jun 29;14:68. doi: 10.1038/s41413-026-00545-1 (PMC13314963; doi:10.1038/s41413-026-00545-1)

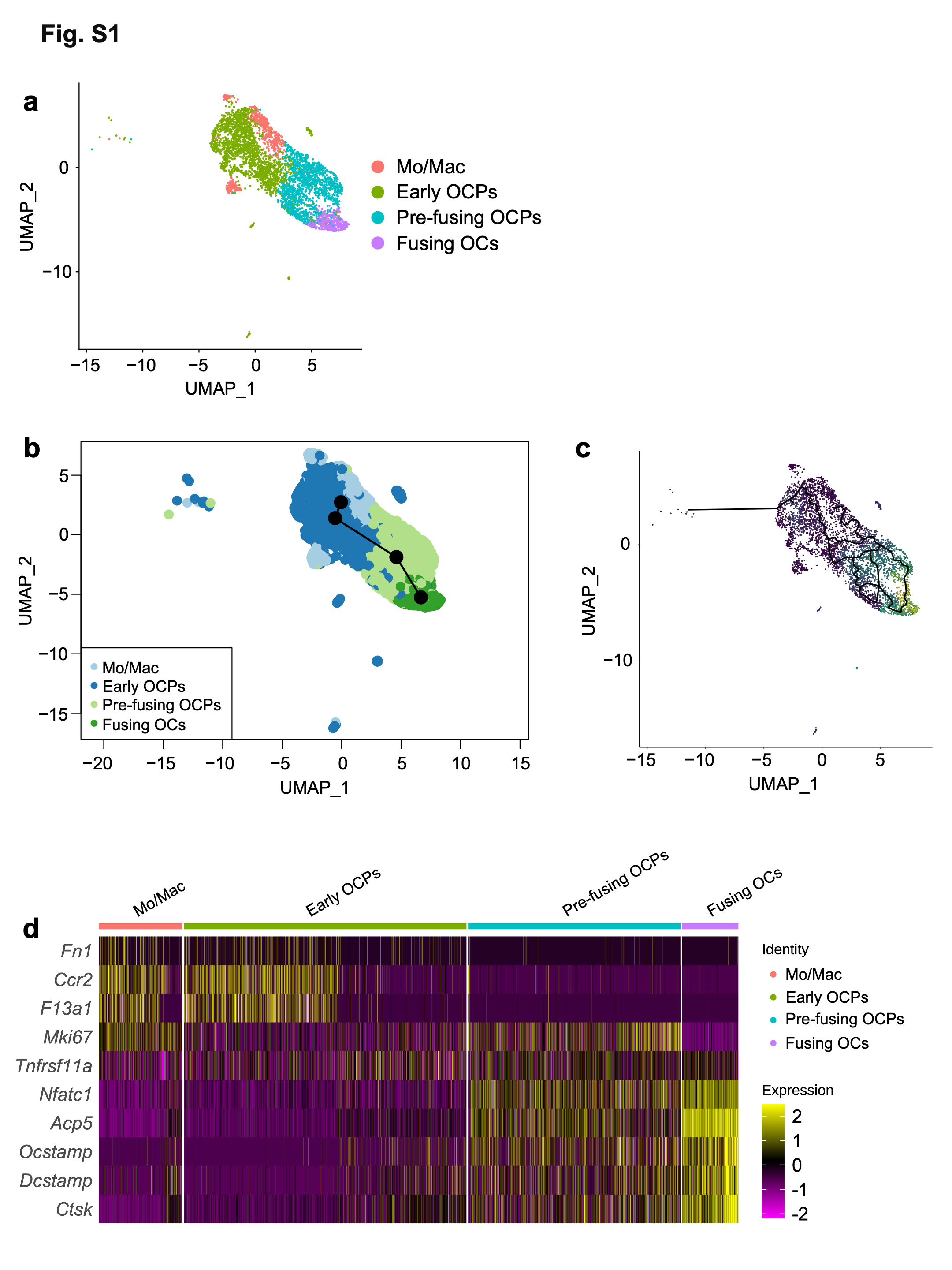

Supplement: Supplementary file 1 — Supplementary Figure S1 [file 41413_2026_545_MOESM1_ESM.tif]

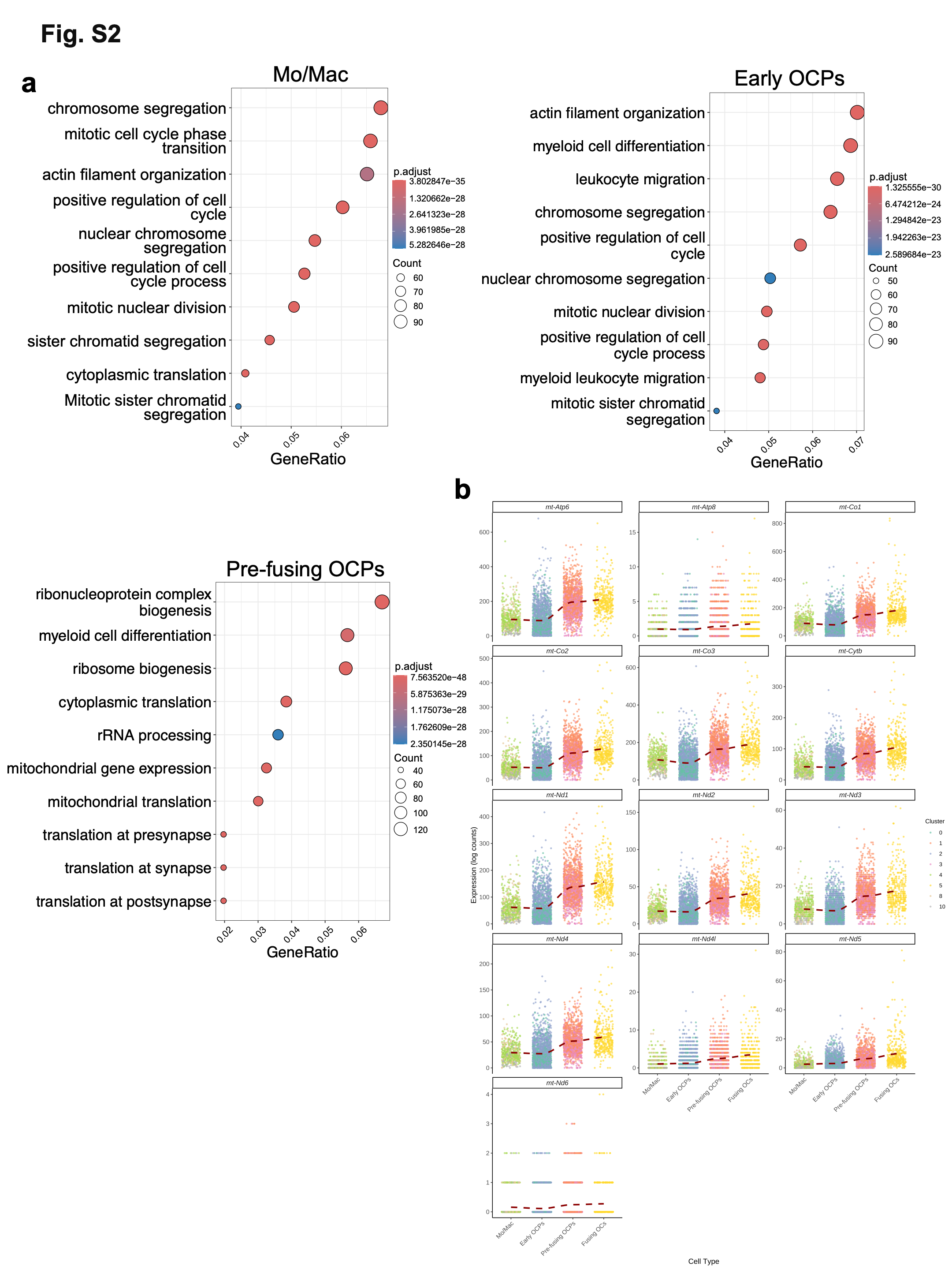

Supplement: Supplementary file 2 — Supplementary Figure S2 [file 41413_2026_545_MOESM2_ESM.tif]

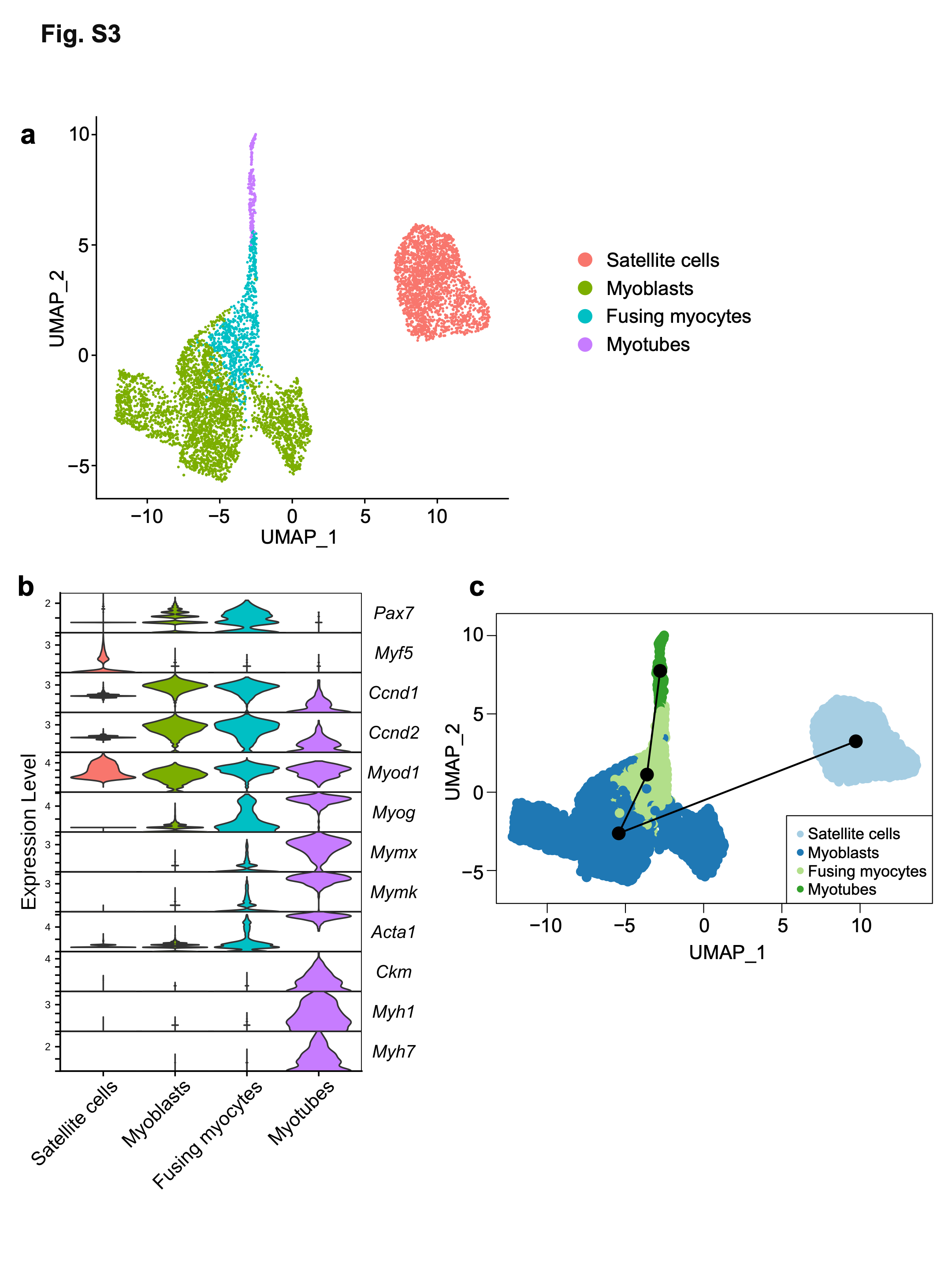

Supplement: Supplementary file 3 — Supplementary Figure S3 [file 41413_2026_545_MOESM3_ESM.tif]

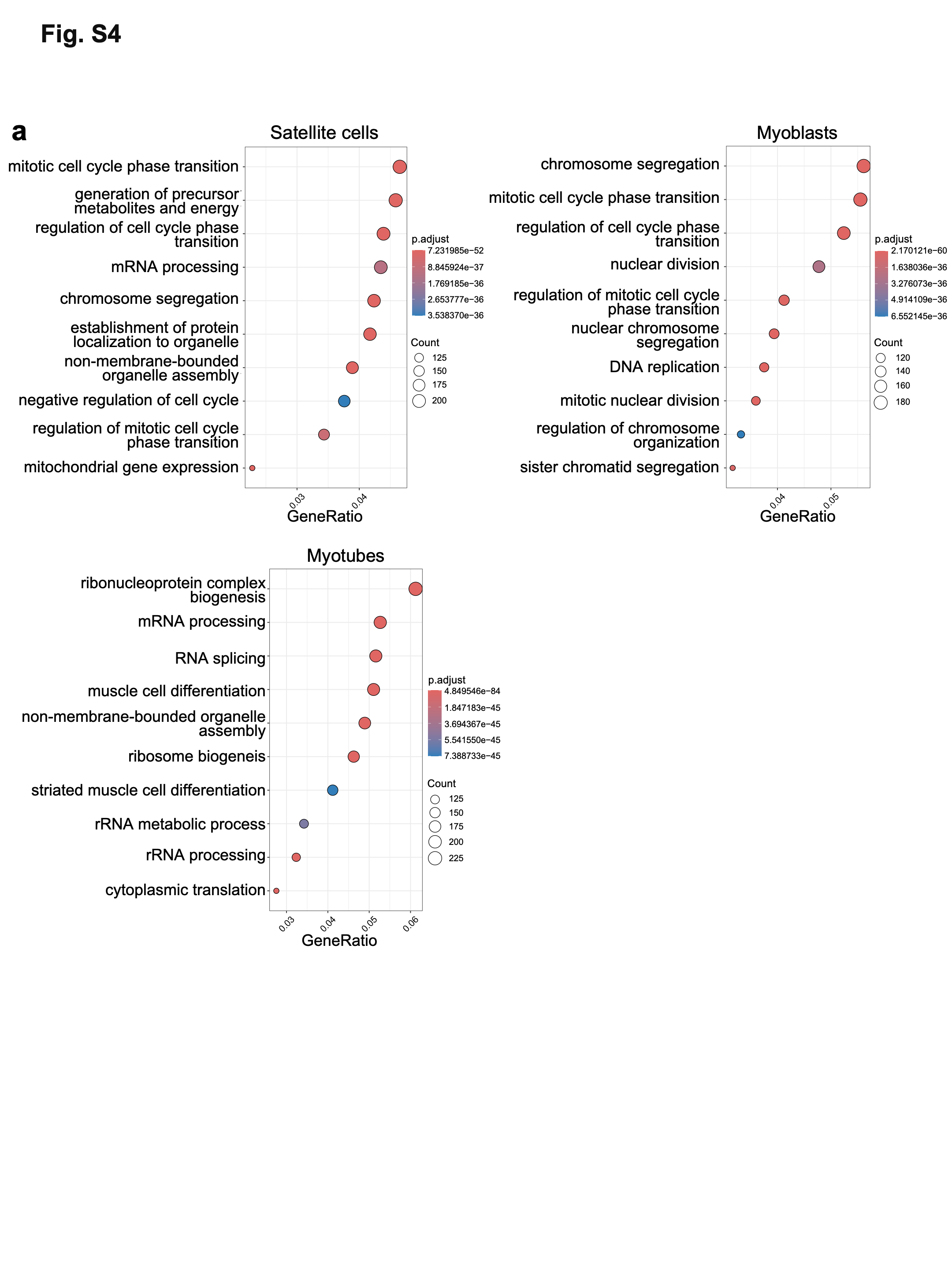

Supplement: Supplementary file 4 — Supplementary Figure S4 [file 41413_2026_545_MOESM4_ESM.tif]

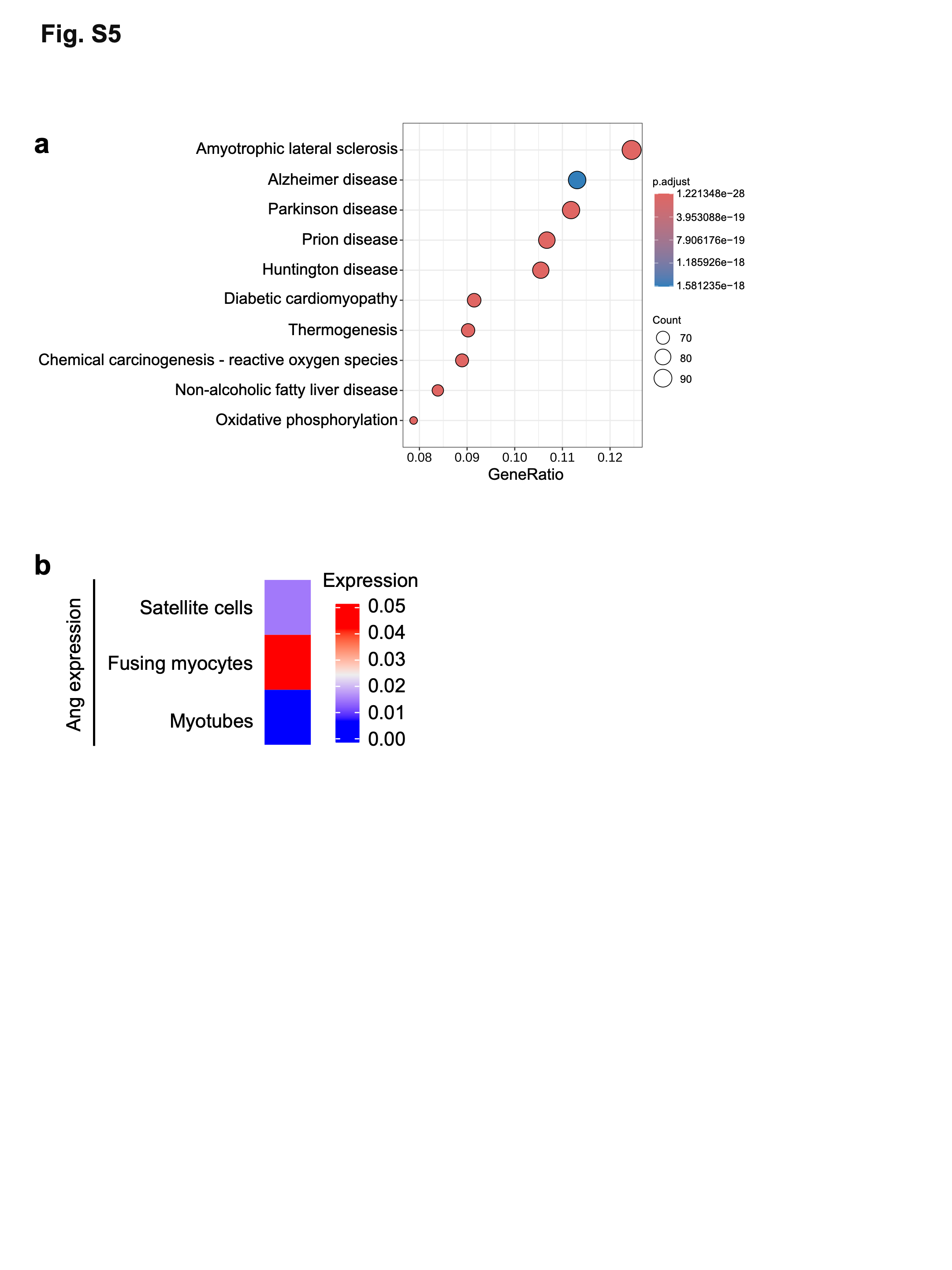

Supplement: Supplementary file 5 — Supplementary Figure S5 [file 41413_2026_545_MOESM5_ESM.tif]

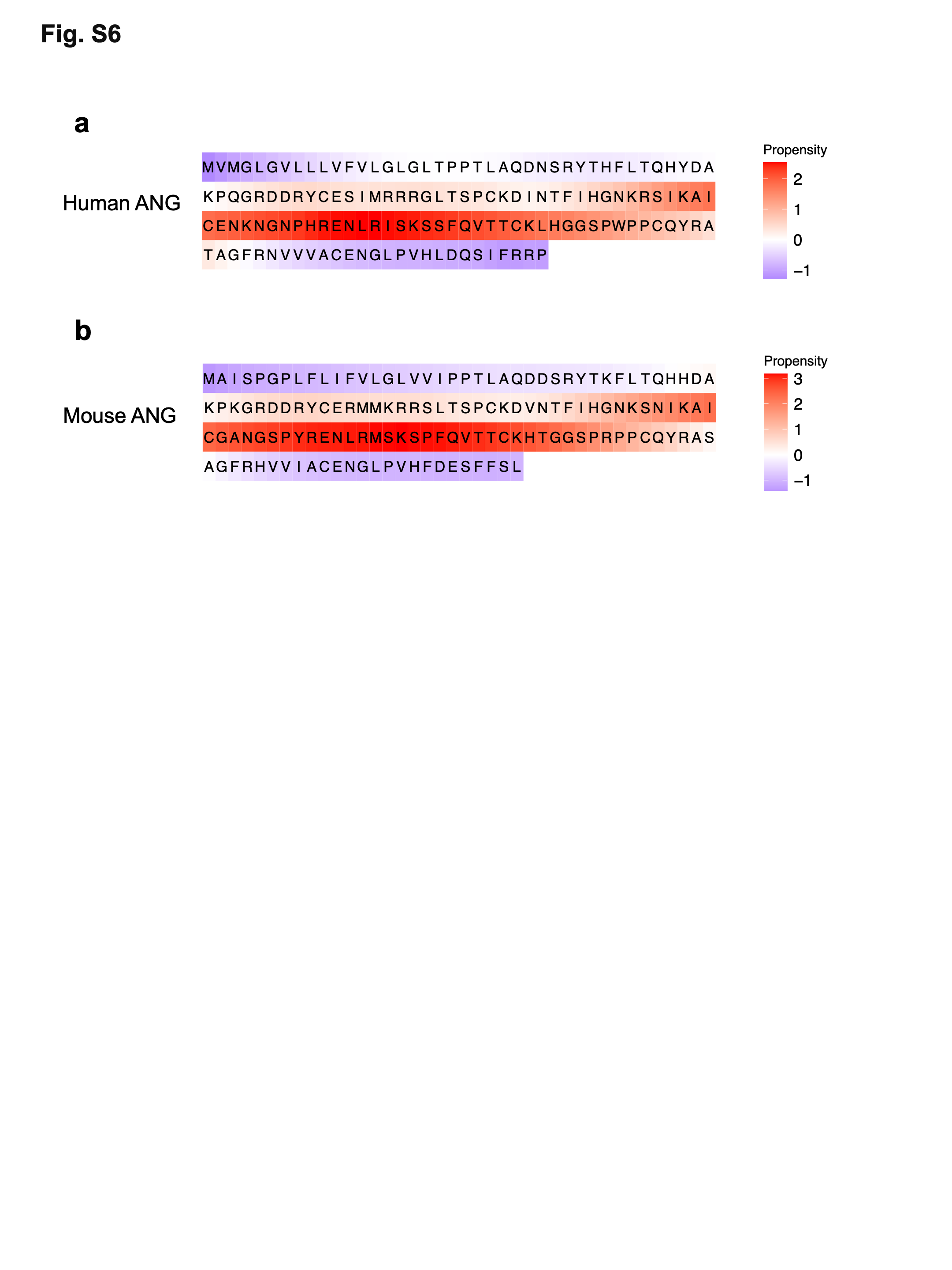

Supplement: Supplementary file 6 — Supplementary Figure S6 [file 41413_2026_545_MOESM6_ESM.tif]

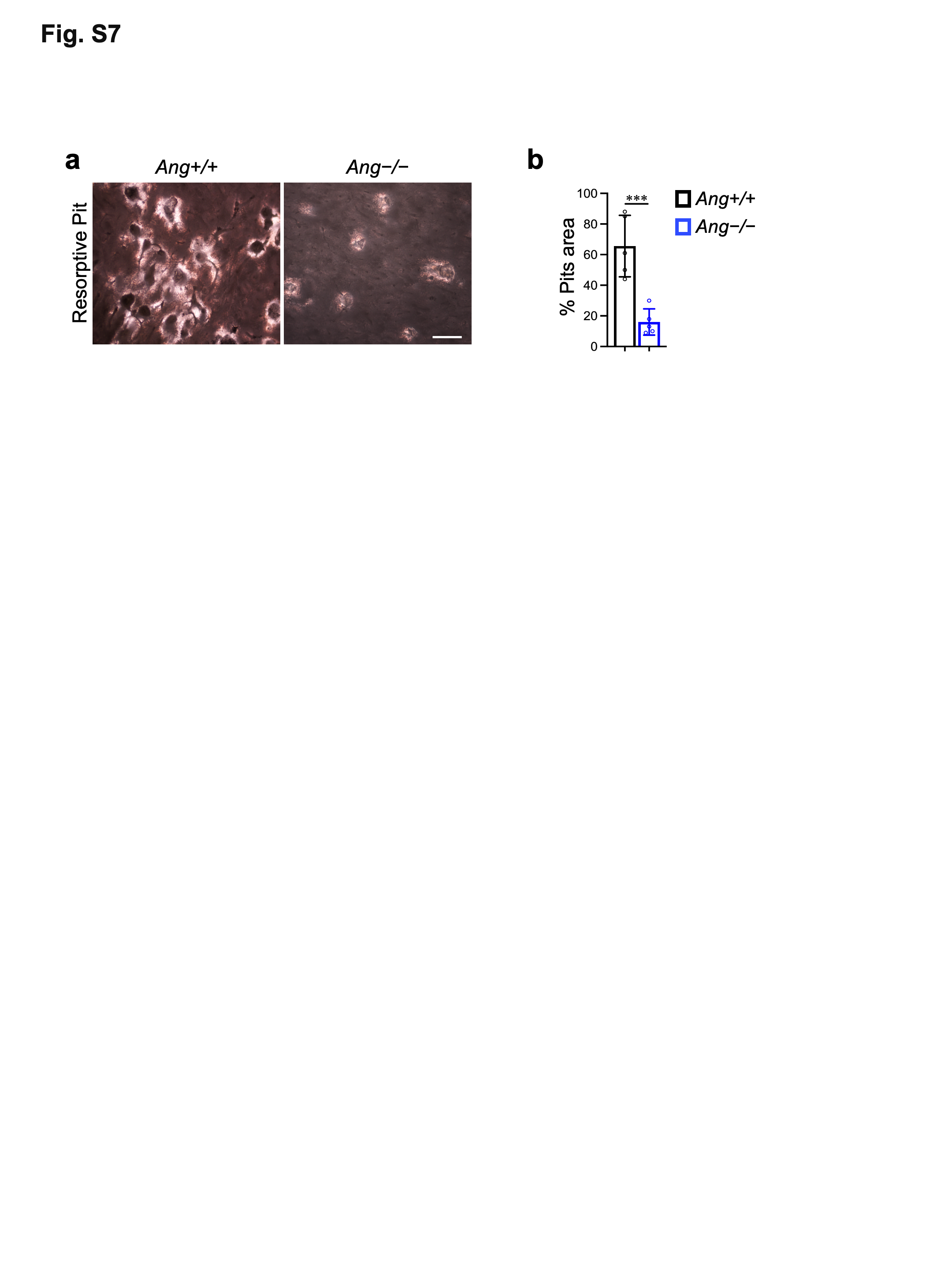

Supplement: Supplementary file 7 — Supplementary Figure S7 [file 41413_2026_545_MOESM7_ESM.tif]

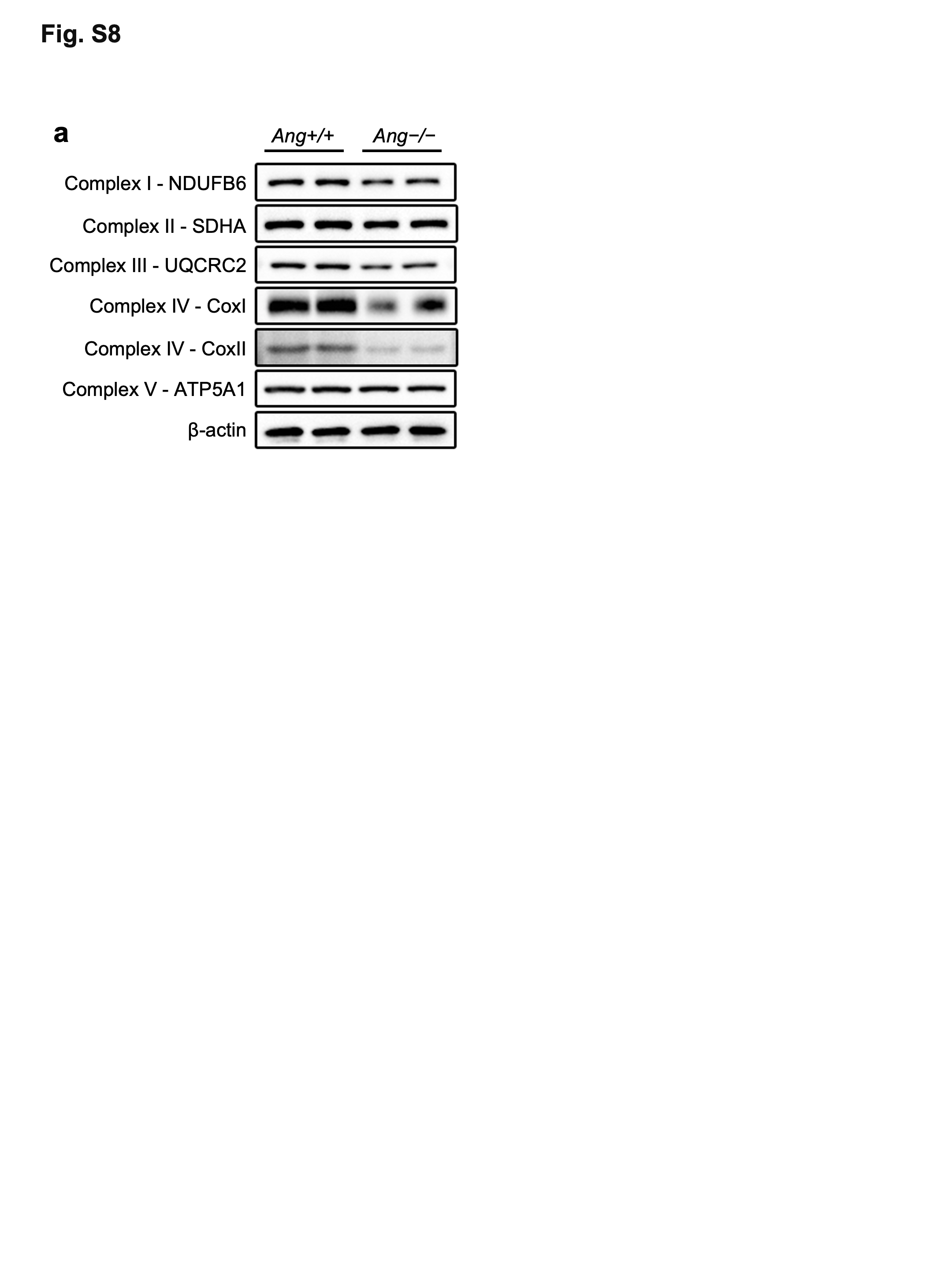

Supplement: Supplementary file 8 — Supplementary Figure S8 [file 41413_2026_545_MOESM8_ESM.tif]

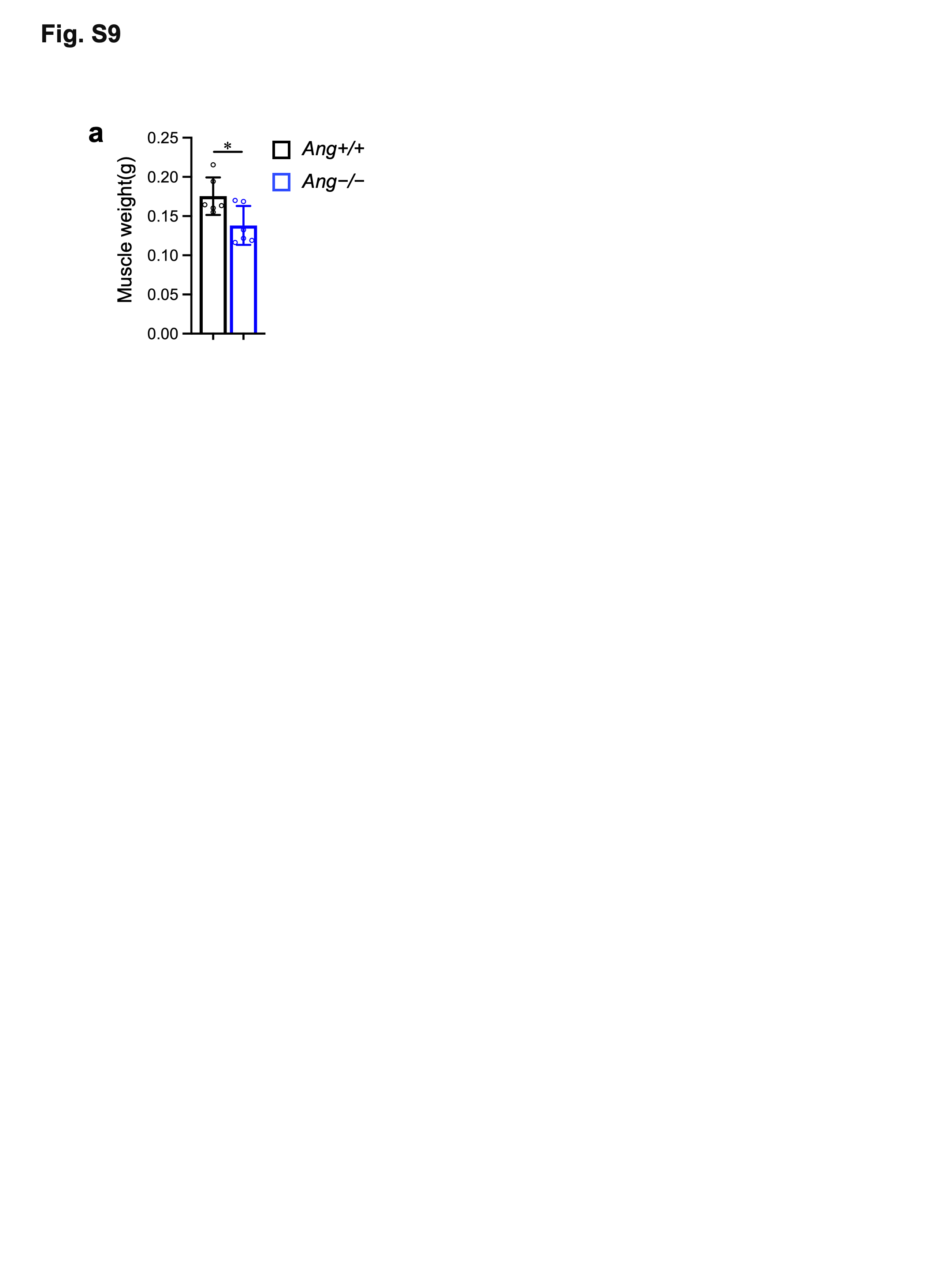

Supplement: Supplementary file 9 — Supplementary Figure S9 [file 41413_2026_545_MOESM9_ESM.tif]

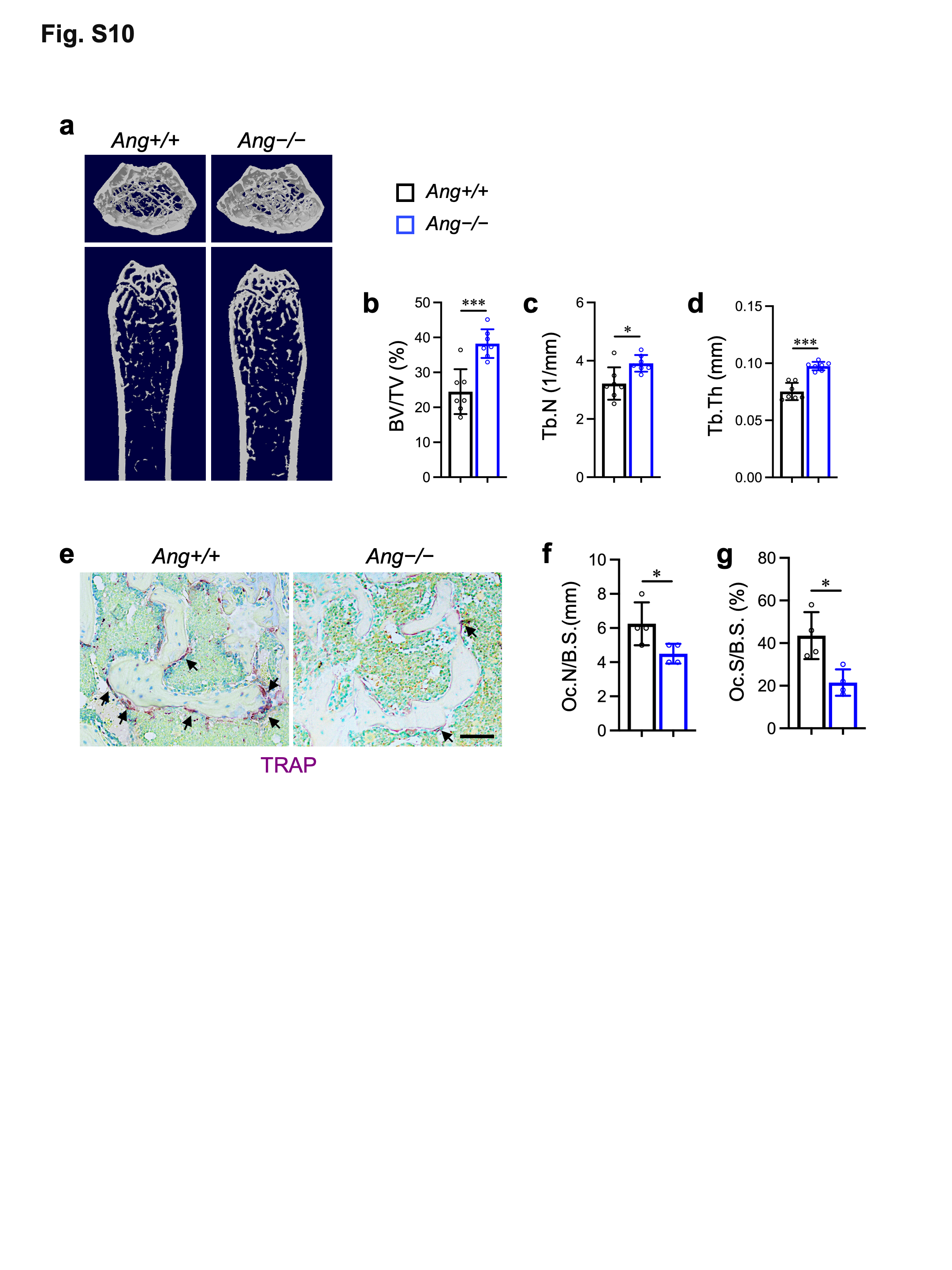

Supplement: Supplementary file 10 — Supplementary Figure S10 [file 41413_2026_545_MOESM10_ESM.tif]

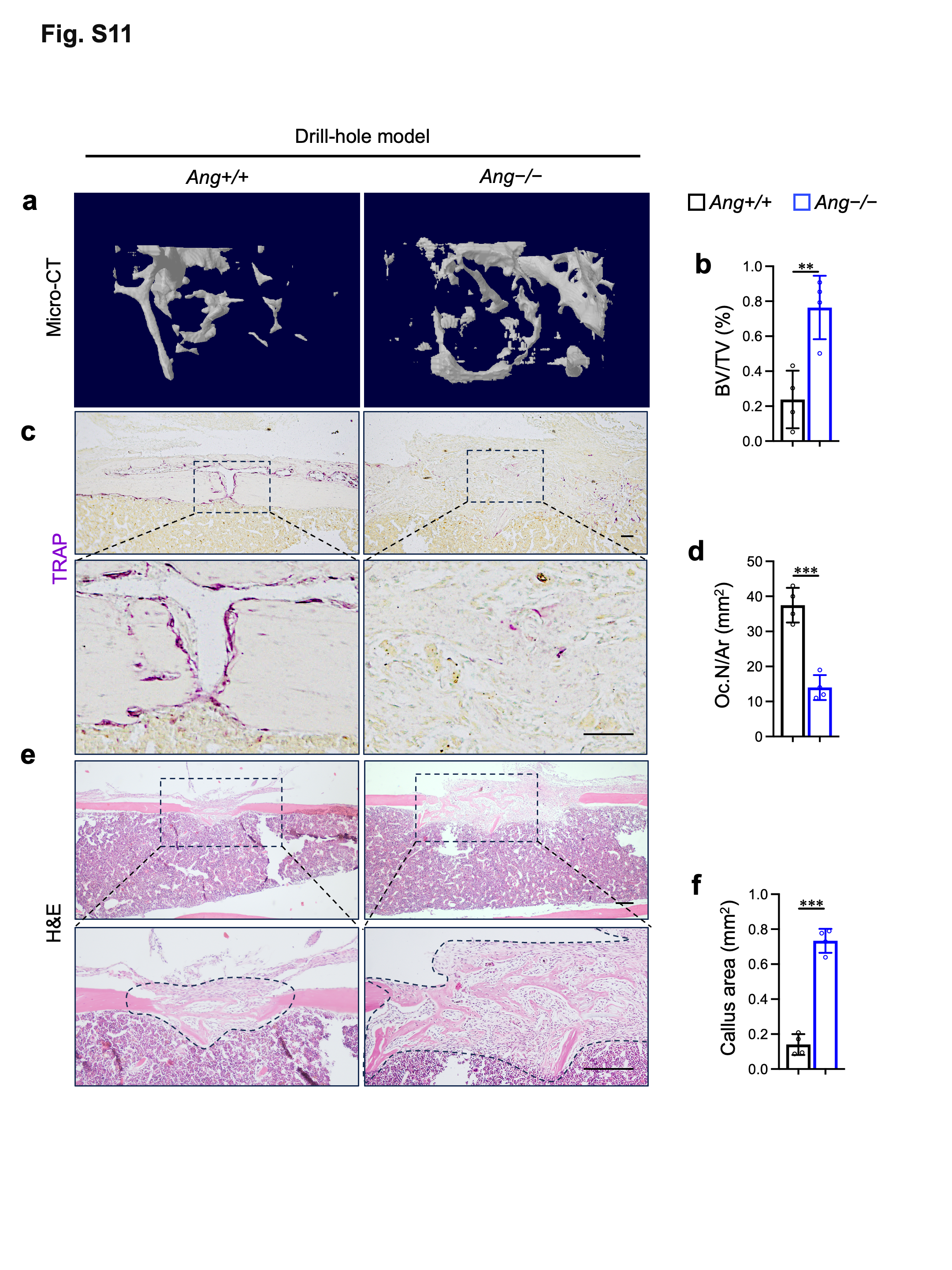

Supplement: Supplementary file 11 — Supplementary Figure S11 [file 41413_2026_545_MOESM11_ESM.tif]

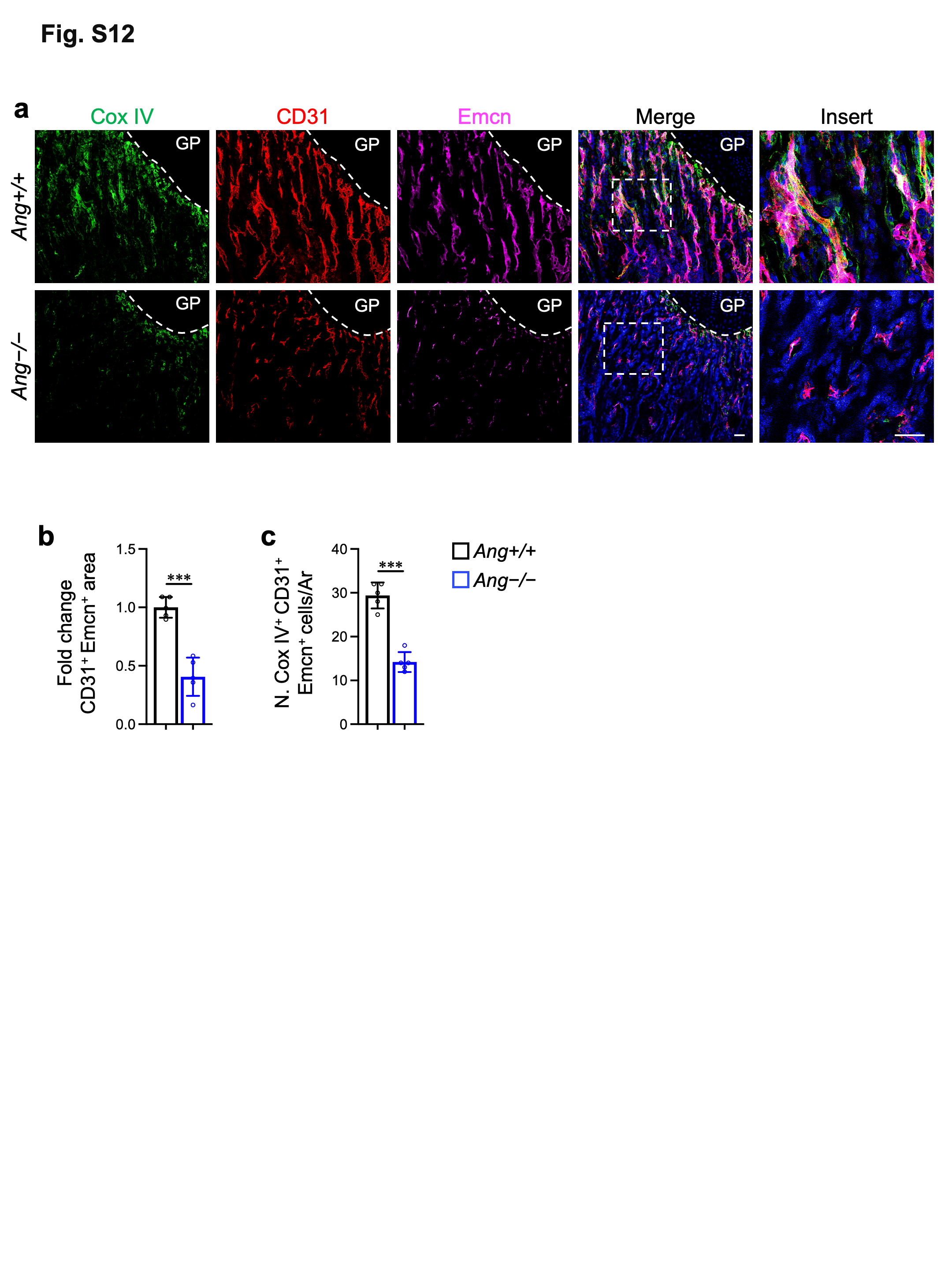

Supplement: Supplementary file 12 — Supplementary Figure S12 [file 41413_2026_545_MOESM12_ESM.tif]

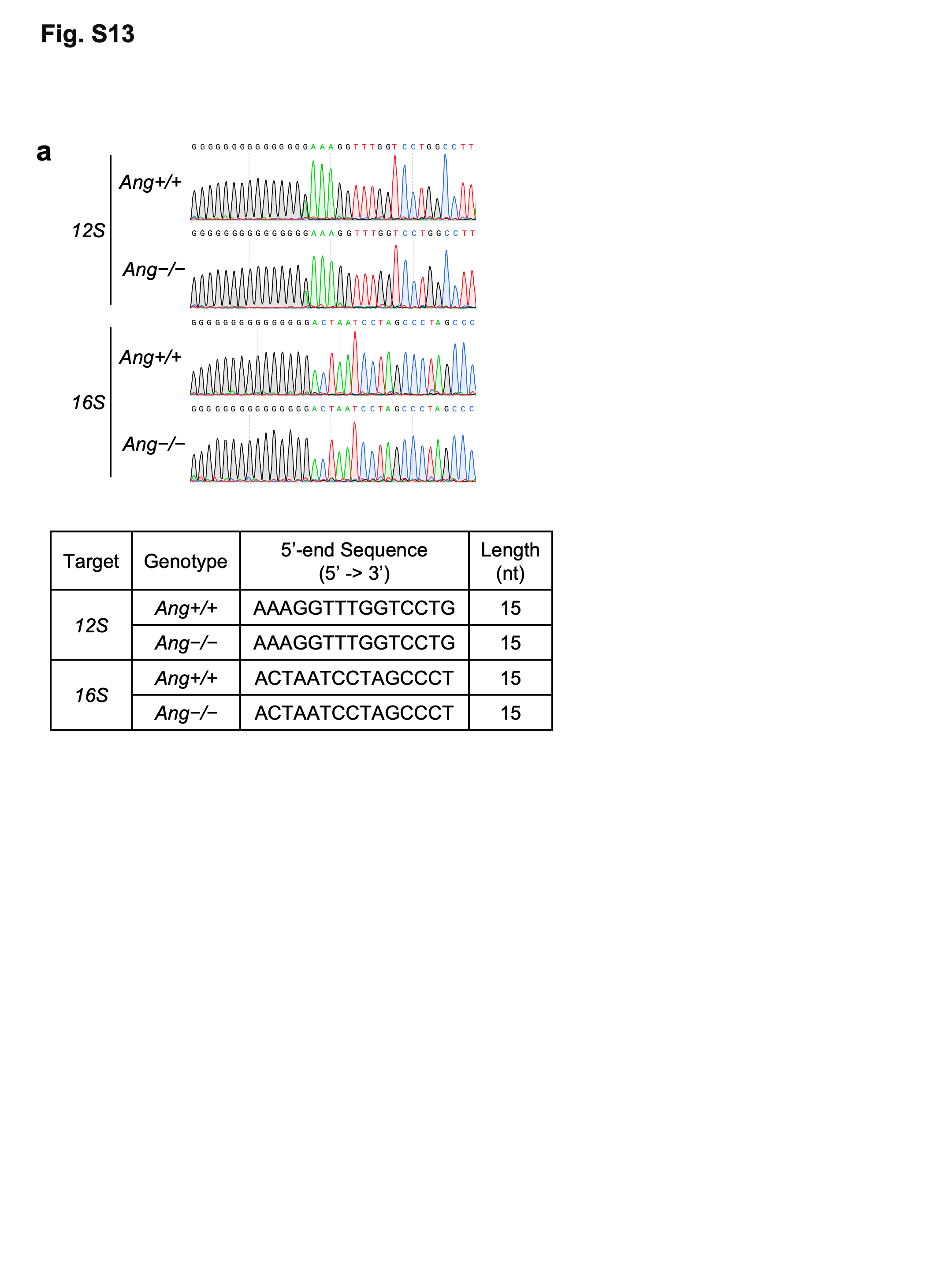

Supplement: Supplementary file 13 — Supplementary Figure S13 [file 41413_2026_545_MOESM13_ESM.tif]

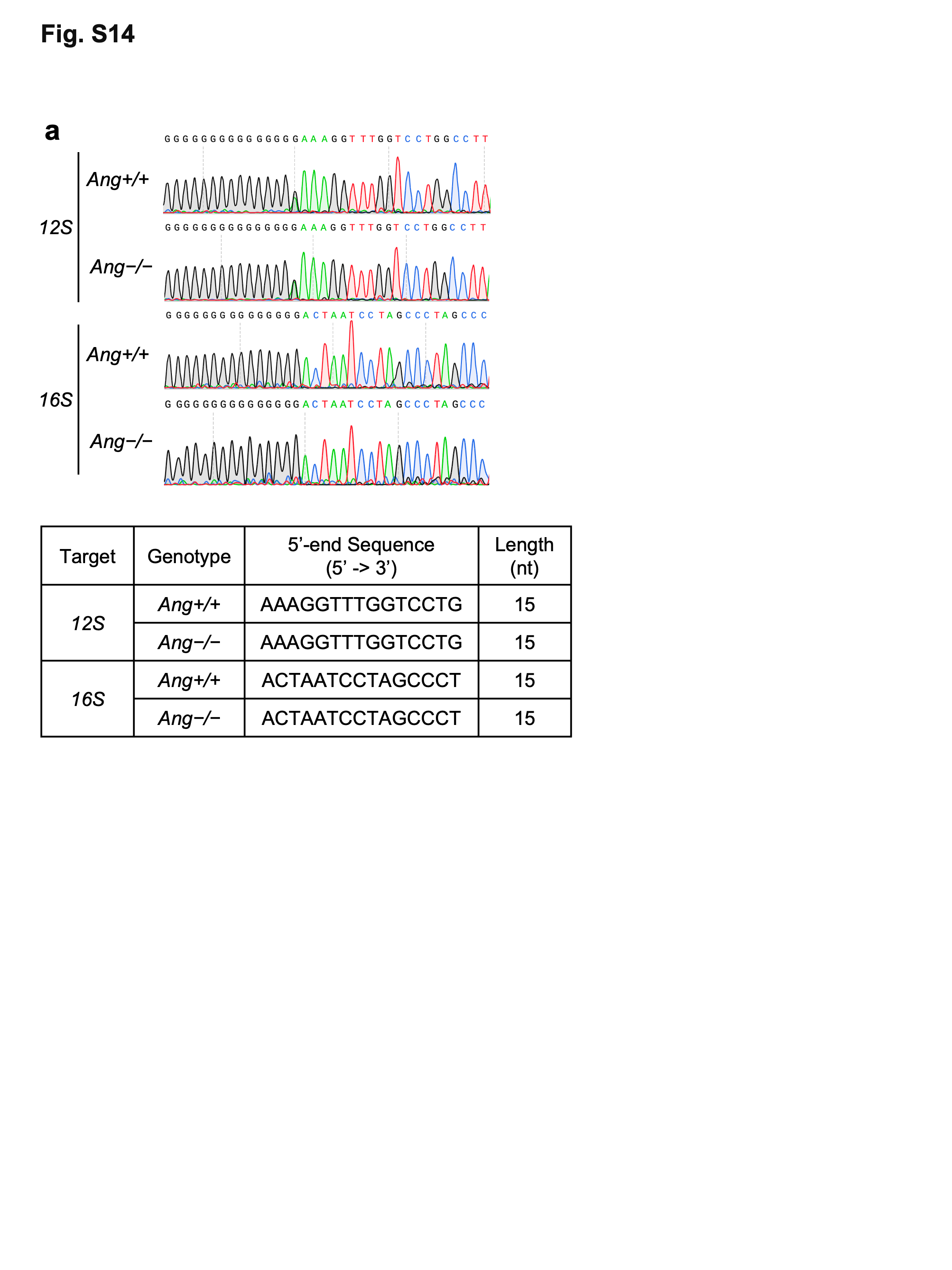

Supplement: Supplementary file 14 — Supplementary Figure S14 [file 41413_2026_545_MOESM14_ESM.tif]

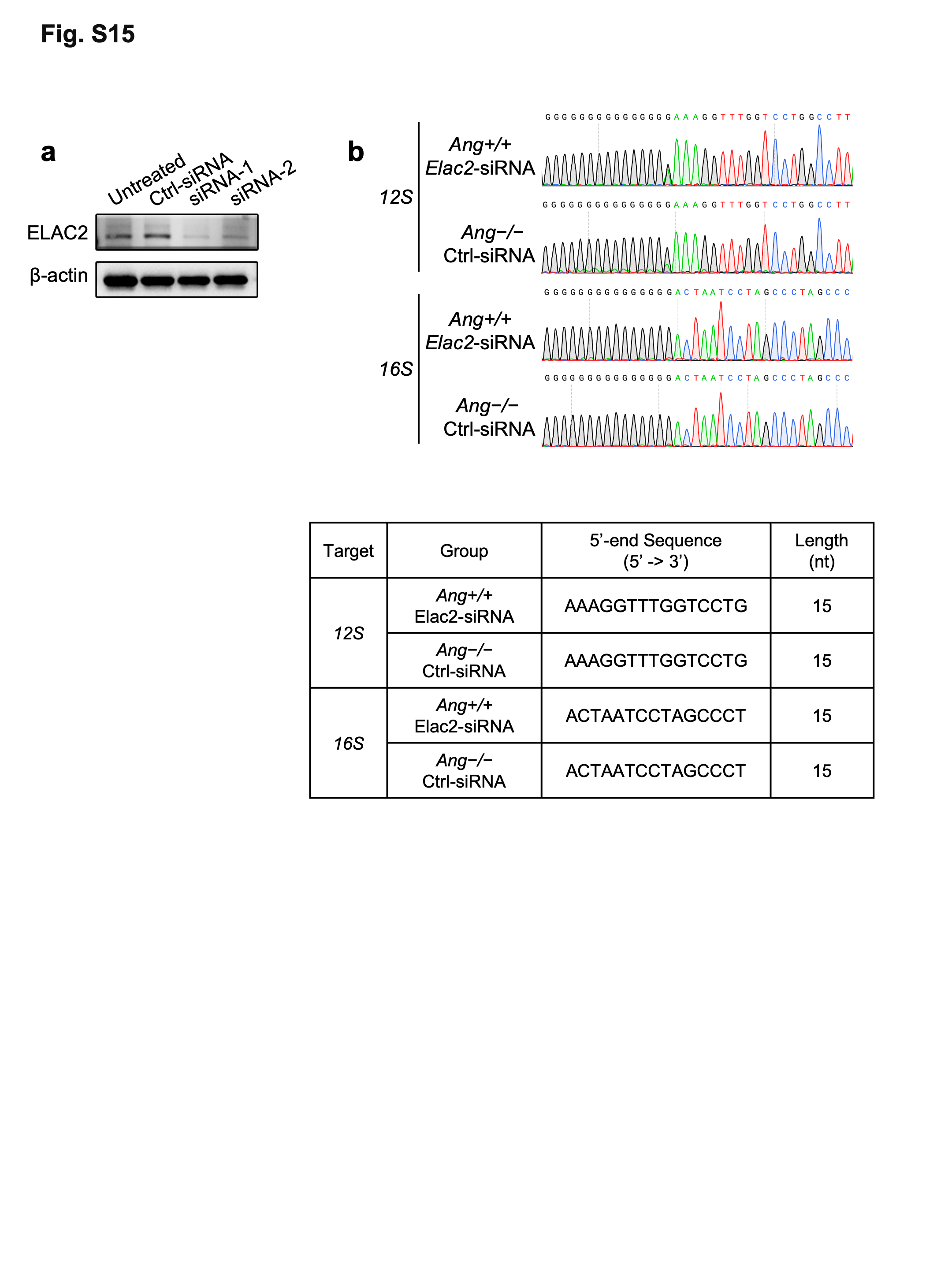

Supplement: Supplementary file 15 — Supplementary Figure S15 [file 41413_2026_545_MOESM15_ESM.tif]

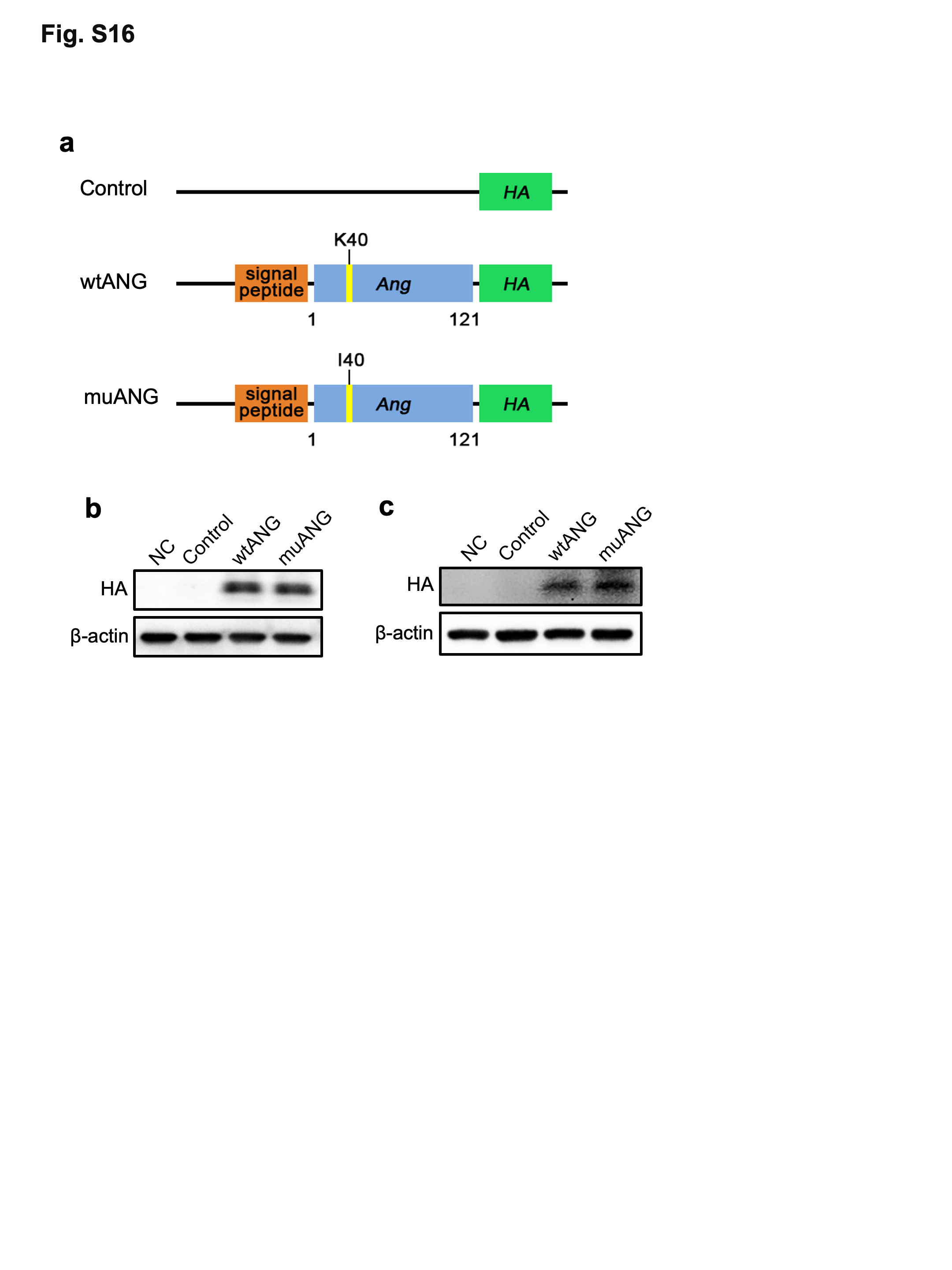

Supplement: Supplementary file 16 — Supplementary Figure S16 [file 41413_2026_545_MOESM16_ESM.tif]
