# Supplementary material for: Angiogenin mediates cell-cell fusion as a mitochondrial RNA processing enzyme
Source: Bone Res. 2026 Jun 29;14:68. doi: 10.1038/s41413-026-00545-1 (PMC13314963; doi:10.1038/s41413-026-00545-1)
Supplement: Supplementary file 19 — Supplementary information [file 41413_2026_545_MOESM19_ESM.docx]

**Fig. S1 Annotation of each stage of osteoclastogenesis by analysis of single-cell RNA-seq dataset.**

**a**, The reanalysis of the published cultured osteoclasts scRNA-seq dataset (GSE147174) was performed. The 13 cell clusters obtained after dimensionality reduction, were annotated and aggregated based on the markers provided in the original work. A uniform manifold approximation and projection (UMAP) visualization of the 8 cell type clusters in the osteoclast culture system was generated. Due to the filtering conditions of Cell Ranger, some cells from the original work were removed, including mature osteoclasts.

**b**, A UMAP plot of of osteoclast lineages is shown, with the branching pseudotime trajectory identified using Slingshot depicted as a black line.

**c**, A UMAP plot embedding colored by pseudotime, illustrating the continuous progression of cells along the inferred trajectory. Dark-to-light coloring reflects increasing pseudotime, as determined by Monocle3 analysis.

**d**, A heatmap is shown, displaying the expression of osteoclast differentiation maker genes in various celltypes.

**Fig. S2 Identification of GO pathways in each stage of osteoclastogenesis by analysis of single cell-Seq dataset.**

**a,** The top 10 GO terms that were enriched in Mo/Mac, early osteoclast precursors (Early OCPs) and pre-fusing osteoclast precursors (Pre-fusing OCPs) are shown.

**b,** The expression of mitochondrial-encoded genes in Mo/Mac, Early OCPs, Pre-fusing OCPs, and Fusing OCs are shown.

**Fig. S3 Annotation each stage of myogeneis by analysis of single-cell RNA-seq dataset.**

**a**, The reanalysis of the published skeletal muscle cells scRNA-seq dataset (GSE126834) was performed. 5 major celltypes obtained after dimensionality reduction, were annotated based on the markers provided in the original work. A uniform manifold approximation and projection (UMAP) visualization of the 5 cell type clusters was generated.

**b**, The stacked violin plot is shown, illustrating the expression of marker genes across various celltypes.

**c**, A UMAP plot of muscle lineages is shown, with the branching pseudotime trajectory identified using Slingshot depicted as a black line.

**Fig. S4 Identification of GO pathways in each stage of myogeneis by analysis of single cell-Seq dataset.**

**a**, The top 10 GO terms that were enriched in satellite cells, myoblasts, and myotubes are shown.

**Fig. S5 Identification of the key gene regulates cell fusion.**

**a**, The top 10 KEGG terms enriched in fusing myocytes are shown.

**b**, The expression of *Ang* in various celltypes is shown in heatmap.

**Fig. S6 ANG contains putative internal matrix targeting-like sequence.**

**a**,**b**, Diagram shown the entire amino acid sequences of human (**a**) and mouse (**b**) ANG. The predicted internal matrix targeting-like sequence (iMTS-Ls) is in red.

**Fig. S7 Loss of ANG impairs bone resoprtion.**

**a**,**b**, One-month-old *Ang* deficient (*Ang*−/−) mice and their wild-type littermates (*Ang+/+*) were used. Mo/Mac were isolated from the mice and cultured in the presence of M-CSF (30 ng/ml) and RANKL (100 ng/ml) for 4 days to acquire mature osteoclasts. Pit resorption assay was performed at 10-day treatment (**a**). Quantification of the relative resorbed pit area were shown in **b**. n= 5 samples per group, and 3 fields per sample were calculated.

Data are shown as mean ± s.d. and analyzed by unpaired Student’s *t* test. ****P* < 0.001.

**Fig. S8 ANG regulates the expression of mitochondrial genes.**

**a**, Bone marrow Mo/Mac from *Ang+/+* and *Ang*−/− mice were treated with M-CSF (30 ng/ml) and RANKL (100 ng/ml) for 3 days to acquire fusing OCs. Western blot analyses of the indicated mitochondria proteins. β-actin expression serves as loading control.

**Fig. S9 The wet weight of skeletal muscle is reduced in *Ang* deficient mice**

**a**, The GA muscles were harvest from 4-month-old *Ang* deficient (*Ang*−/−) mice and their wild-type littermates (*Ang+/+*). Quantified analysis of the wet weight of individual muscles were shown in **a**.

Data are shown as mean ± s.d. and analyzed by unpaired Student’s *t* test. **P* < 0.05.

**Fig. S10 Loss of ANG leads to impaired bone resorption in 4-month-old mice**

**a**–**d**, Bone phenotype of 4-month-old *Ang*−/− mice and their wild-type littermates (*Ang+/+*) were assessed. Micro-CT analysis of distal femurs of the mice. Representative images of trabecular region were shown in **a**. Upper, trabecular cross sections; lower, longitudinal sections. Quantitative analyses of trabecular bone volume (BV/TV) (**b**), trabecular number (Tb. N) (**c**), and trabecular thickness (Tb. Th) (**d**). n=7 mice per group.

**e**–**g**, TRAP staining of femoral bone tissue sections. Representative images were shown in **e**, and the calculation of osteoclast number (Oc.N/B.S.) and osteoclast surface (Oc.S/B.S.) per bone surface were shown in **f** and **g**, respectively. Scale bars, 50 μm. n=4.

Data are shown as mean ± s.d. and analyzed by unpaired Student’s t test. ****P* < 0.001, and **P* < 0.05.

**Fig. S11 Loss of ANG leads to impaired callus removal.**

Drill-hole bone defects were created in 10-week-old *Ang−/−* mice and their wild-type littermates (*Ang+/+*). Bone tissues were collected 14 days following injury.

**a,b**, Micro-CT analysis callus size in the defect sites of the mice. Representative images of trabecular region were shown in (**a**). Quantitative analyses of trabecular bone volume (BV/TV%) (**b**) n=4 mice per group.

**c,d**, TRAP staining of bone sections to show osteoclasts. Representative images (**c**) and quantitative analysis (**d**) are shown. Scale bars, 50 μm. n=4.

**e,f**, H&E staining of bone sections to show callus size and structure. Representative images (**e**) and quantitative analysis (**f**) are shown. Scale bars, 200 μm. n=4.

Data are shown as mean ± s.d. and analyzed by unpaired Student’s *t* test. ****P* < 0.001, and ***P* < 0.01.

**Fig. S12 Loss of ANG leads to mitochondrial dysfunction in type-H vessels.**

**a**–**c**, Immunofluorescence staining of femur sections from 1-month-old *Ang+/+* and *Ang*−/− mice was performed using antibodies against CoxIV (green), CD31 (red), and Emcn (magenta). Representative images are shown in (**a**), and the fold change of the CD31^+^ Emcn^+^ overlapping area and the number of CoxIV^+^ CD31^+^ Emcn^+^ cells per mm^2^ tissue area are shown in **b** and **c,** respectively. Scale bars, 40 μm. n=5.

Data are shown as mean ± s.d. and analyzed by unpaired Student’s t test. ****P* < 0.001.

**Fig. S13 *Ang* deficiency does not affect mitochondrial RNA processing in Mo/Mac without RANKL stimulation.**

**a**, Mouse Mo/Mac from *Ang+/+* and *Ang*−/− mice were cultured in medium containing M-CSF (30 ng/ml). Mitochondrial RNA from the cells were subjected to 5’RACE. Sanger DNA sequences of 5’RACE products for 5’end of *12S* and *16S*. The first 15 nucleotides (nt) of the *12S* and *16S* sequences are shown in the table.

**Fig. S14 Sanger DNA sequencing results of 5’RACE products in differentiating osteoclasts.**

**a**, Sanger DNA sequence for 5’ end of *12S* and *16S* in *Ang+/+* and *Ang*−/− fusing OCs. The first 15 nucleotides (nt) of the *12S* and *16S* sequences are shown in the table.

**Fig. S15 ANG independently cleaves tRNA 3’ end during osteoclastogenesis.**

**a**, Mouse bone marrow Mo/Mac from *Ang+/+* and *Ang−/−* mice transfected with individual siRNAs: negative control siRNA (Ctrl-siRNA), *Elac2* siRNA-1 (siRNA-1), and *Elac2* siRNA-2 (siRNA-2). Three days after infection, western blot was conducted to detect the RNase Z (*Elac2* encoded protein) expression in the cells.

**b**, Mouse bone marrow Mo/Mac from *Ang+/+* and *Ang−/−* mice were transfected with individual siRNAs as indicated followed by RANKL (100ng/ml) for 3 days to acquire fusing OCs. Mitochondrial RNA from the cells were subjected to 5’RACE. The corresponding PCR products were sequenced to confirm their 5’ end sequences. The first 15 nucleotides (nt) corresponding to 5’ end of *12S* and *16S* sequences are shown in the table.

**Fig. S16 Generation and validation of the viruses carrying wild-type and mutant ANG.**

**a**, Schematic diagram shows the insertion sequences of the lentiviruses carrying empty vector (Control), wild-type *Ang* (wtANG), and mutant *Ang* (muANG).

**b**,**c**, Skeletal muscle satellite cells (**b**) and Mo/Mac (**c**) isolated from *Ang+/+* and *Ang−/−* mice were infected with individual lentiviruses as indicated. 3 days after infection, western blot was conducted to detect the HA-tag expression in the cells.

**Supplementary Table 1. Statistics of 5’- and 3’-end cleavage of mt-tRNA. (Separate Excel file is attached)**

**Supplementary Table 2. Frequency of 5’- and 3’-end cleavage of mt-tRNA. (Separate Excel file is attached)**

**Supplementary Table 3. Quality control of PARE-Seq.**

| **Quality control of PARE-Seq** | | | | |
| --- | --- | --- | --- | --- |
| Sample | KO(number) | KO(ratio) | WT(number) | WT(ratio) |
| Raw Reads | 9947699 | / | 10909608 | / |
| Unique Raw Reads | 3695668 | / | 3674218 | / |
| reads < 15nt after removing 3 adaptor | 806023 | 8.10% | 727979 | 6.67% |
| Mappable Reads | 9141676 | 91.90% | 10181629 | 93.33% |
| Unique reads < 15nt after removing 3 adaptor | 78336 | 2.12% | 67887 | 1.85% |
| Unique Mappable Reads | 3617332 | 97.88% | 3606331 | 98.15% |
| Mapped Reads | 2015167 | 20.26% | 1938044 | 17.76% |
| Unique Mapped Reads | 682399 | 18.46% | 638030 | 17.37% |
| Number of input Transcript | 133520 | / | 133520 | / |
| Number of Coverd Transcript | 56565 | 42.36% | 54538 | 40.85% |

**Supplementary Table 4. Primer sequences for mouse qRT-PCR and 5’ RACE assay.**

| **Mouse qRT-PCR and 5’ RACE assay Primer sequences** | |
| --- | --- |
| **Primer** | **Sequence** |
| β-Actin-F | GACGGCCAGGTCATCACTATTG |
| β-Actin-R | AGGAAGGCTGGAAAAGAGCC |
| TRNF-RNR1-F | AGCACTGAAAATGCTTAGATGG |
| TRNF-RNR1-R | TAATTATAAGGCCAGGACCAAACC |
| 12SrRNA_5F | CACGGGACTCAGCAGTGATA |
| 12SrRNA_5R | GAGATGGTGAGGTAGAGCGG |
| RNR1-TRNV-F | CATACTGGAAAGTGTGCTTGGA |
| RNR1-TRNV-R | TCTTCTGGGTGTAGGCCAGAT |
| TRNV-RNR2-F | TCTGGCCTACACCCAGAAGA |
| TRNV-RNR2-R | TGTGTAGGGCTAGGGCTAGGA |
| 16SrRNA_3F | TGACCGTGCAAAGGTAGCAT |
| 16SrRNA_3R | TATTCTCCGAGGTCACCCCA |
| RNR2-TRNL1-F | ACCTTACAAATAAGCGCTCTCAA |
| RNR2-TRNL1-R | TAACCTTCTCTAGGTTAGAGGGTG |
| ND1-F | TGCACCTACCCTATCACTCA |
| ND1-R | GGCTCATCCTGATCATAGAATGG |
| ND2-F | ATACTAGCAATTACTTCTATTTTCATAGGG |
| ND2-R | GAGGGATGGGTTGTAAGGAAG |
| ND3-F | AAGCAAATCCATATGAATGCGG |
| ND3-R | GCTCATGGTAGTGGAAGTAGAAG |
| ND4-F | CATCACTCCTATTCTGCCTAGC |
| ND4-R | CCAACTCCATAAGCTCCATACC |
| COX1-F | CCCAGATATAGCATTCCCACG |
| COX1-R | ACTGTTCATCCTGTTCCTGC |
| COX2-F | AGTTGATAACCGAGTCGTTCTG |
| COX2-R | CTGTTGCTTGATTTAGTCGGC |
| COX3-F | CGTGAAGGAACCTACCAAGG |
| COX3-R | CGCTCAGAAGAATCCTGCAA |
| ATP8-F | GCCACAACTAGATACATCAACATG |
| ATP8-R | TGGTTGTTAGTGATTTTGGTGAAG |
| ATP6-F | TCCCAATCGTTGTAGCCATC |
| ATP6-R | TGTTGGAAAGAATGGAGTCGG |
| ND4L-F | CCAACTCCATAAGCTCCATACC |
| ND4L-R | GATTTTGGACGTAATCTGTTCCG |
| ND5-F | ACGAAAATGACCCAGACCTC |
| ND5-R | GAGATGACAAATCCTGCAAAGATG |
| ND6-F | TGTTGGAGTTATGTTGGAAGGAG |
| ND6-R | CAAAGATCACCCAGCTACTACC |
| Ctyb-F | CCCACCCCATATTAAACCCG |
| Ctyb-R | GAGGTATGAAGGAAAGGTATAAGGG |
| 12S-GSP1 | TTGGGTCTTAGCTGTCGTGT |
| 12S-GSP2 | GGCTGGCACGAAATTTACCA |
| 16S-GSP1 | GAGCTGTCCCTCTTTTGGCT |
| 16S-GSP2 | AGCTCGTTAGGCTTTTCACCT |
